# Modular tissue-in-a-CUBE platform to model blood-brain barrier (BBB) and brain interaction

**DOI:** 10.1038/s42003-024-05857-8

**Published:** 2024-02-28

**Authors:** Isabel Koh, Masaya Hagiwara

**Affiliations:** 1 Cluster for Pioneering Research, RIKEN, Kobe, Hyogo Japan; 2 Biosystems Dynamics Research, RIKEN, Kobe, Hyogo Japan

**Keywords:** Lab-on-a-chip, Neurological models, Tissue engineering

## Abstract

With the advent of increasingly sophisticated organoids, there is growing demand for technology to replicate the interactions between multiple tissues or organs. This is challenging to achieve, however, due to the varying culture conditions of the different cell types that make up each tissue. Current methods often require complicated microfluidic setups, but fragile tissue samples tend not to fare well with rough handling. Furthermore, the more complicated the human system to be replicated, the more difficult the model becomes to operate. Here, we present the development of a multi-tissue chip platform that takes advantage of the modularity and convenient handling ability of a CUBE device. We first developed a blood-brain barrier-in-a-CUBE by layering astrocytes, pericytes, and brain microvascular endothelial cells in the CUBE, and confirmed the expression and function of important tight junction and transporter proteins in the blood-brain barrier model. Then, we demonstrated the application of integrating Tissue-in-a-CUBE with a chip in simulating the in vitro testing of the permeability of a drug through the blood-brain barrier to the brain and its effect on treating the glioblastoma brain cancer model. We anticipate that this platform can be adapted for use with organoids to build complex human systems in vitro by the combination of multiple simple CUBE units.

## Abstract (web summary)

Development of platform to integrate multiple Tissue-in-a-CUBEs in a chip for tissue-tissue interaction, demonstrated by simulating the testing of the permeability and effect of a cancer drug in a BBB-Brain cancer model.

## Introduction

Neurological disorders are increasingly becoming contributors to a decreased quality of life in ageing population worldwide^1^. Yet, progress in drug development for neurological diseases is often hampered by difficulties in getting the therapeutic substances past the blood-brain barrier (BBB) into the central nervous system. To overcome these difficulties, human in vitro models of the BBB are highly desired to efficiently conduct experiments to determine the permeability of a drug candidate through the BBB, as well as to study its effect on the brain. Furthermore, BBB models are also useful to test for whether drugs targeting other tissues of interest would enter or affect the brain as a side effect. The main components of the BBB are endothelial cells, pericytes, astrocytes, and a basement membrane that together contribute to maintaining the barrier function and homoeostasis of the brain via tight junction and transporter proteins^2–4^. Given the complex makeup of the BBB, various models have been established with different combinations of cellular and basement membrane components—astrocytes, pericytes and brain endothelial cells from primary, immortalized, or pluripotent stem cell (PSC)-derived sources have been used with synthetic membranes or hydrogels as basement membrane^5–14^.

Besides the cellular and structural components of the BBB, the interaction between the BBB and the brain is also important to be taken into consideration in developing BBB models. In neurodegenerative diseases and in the ageing brain, a cycle of the accumulation of pathological proteins such as amyloid-β in Alzheimer’s disease or Lewy bodies in Parkinson’s disease leading to dysfunction of the BBB, leading to further accumulation of pathological proteins and further disruption of the BBB, is thought to contribute to the progression of the disease^15–18^. Thus, to effectively develop and test drug candidates to treat these diseases requires recapitulating not just the healthy BBB, but also the dynamics of the BBB-brain interaction, particularly in the diseased state.

Organoids derived from stem cells have been shown to mimic some of the function of native organs, and with the rapid expansion of disease-modelling brain organoids being developed^19,20^, there is increasing demand to progress in vitro BBB models to include a brain component to more closely represent in vivo conditions. Combining different tissue models to achieve a more physiological representation of an organ, commonly termed Organ-on-a-Chip systems, can be achieved in two main ways: (i) by simple merging of the separately cultured tissues such as co-culturing retinal organoid on a bed of retinal pigment epithelium (RPE) cells^21^ or brain organoid on a vascular network^22^, or (ii) by connecting organoids cultured in modular components via microfluidic channels, for example by linking multiple Transwell inserts in a gut-liver or 4-organ model^23,24^ or by seeding organoids directly into the chambers of a multi-organ microfluidic device^25^.

Lamentably, many of these engineered models are not widely adopted by biology-based laboratories that study disease mechanisms or drug discovery and development, ostensibly because they do not have the time or resources for the setup processes that can be long and complicated for those unfamiliar with the technologies. Furthermore, in most Organ-on-a-Chip systems, the cells adhere and grow on the device, making it difficult to retrieve the sample for later use or analysis without detaching the cells from the device, which can cause damage to the sample. Hence, there is a need to balance replicating in vivo complexities in the lab, but with simple setup procedures that can be handled by researchers without an engineering background.

**Fig. 1:**
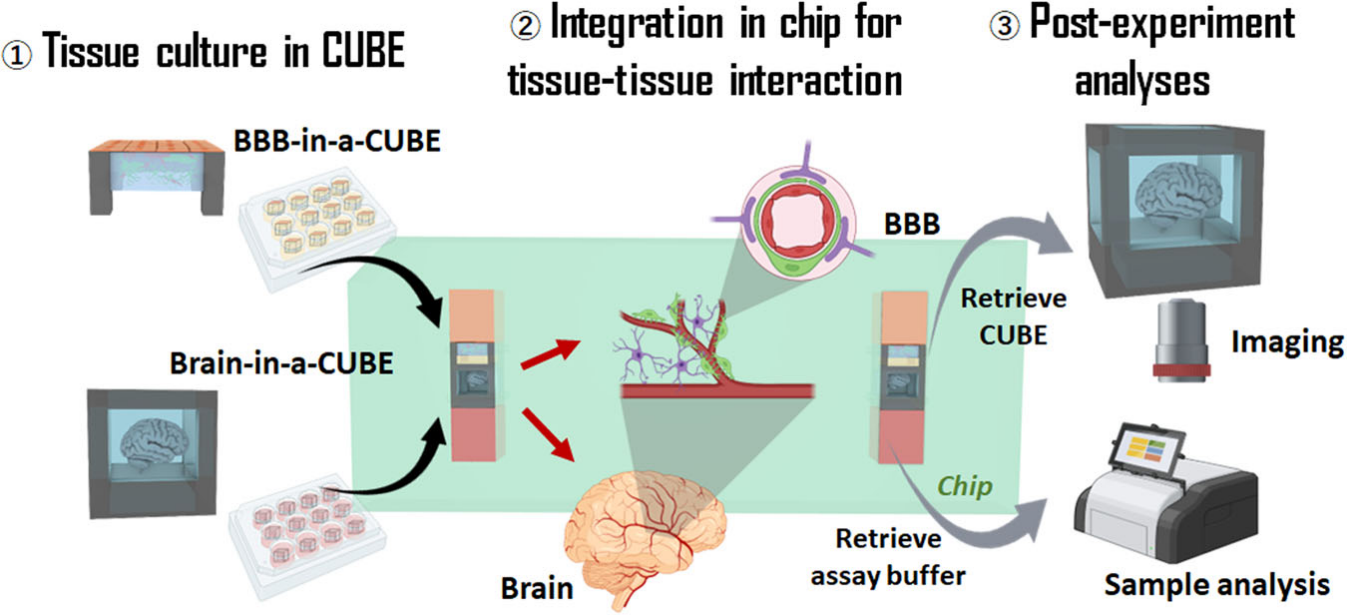
Integration of multiple Tissue-in-a-CUBEs for multi-tissue interaction. The concept of this work was to utilise the CUBE culture device to facilitate the modular combination of complex 3D tissues in simple units to build complex systems. (1) Modular tissue units are first cultured in individual CUBEs, (2) then transferred to a chip at the appropriate timing to initiate tissue-tissue interaction, and (3) at the end of the experiment, samples can be retrieved from the chip for analyses.

In this paper, we present a highly modular and adaptable platform for Organ-on-a-Chip development, which takes advantage of the ease of sample handling of our previously published CUBE culture device^26–28^ to effortlessly integrate multiple separately cultured tissue or organoid models in a simple chip device (Fig. 1). The desired tissue or organoid can be reconstructed in the CUBE with a combination of the appropriate cell types and extracellular matrix (ECM) hydrogel that provides a 3D scaffold for cells. As the cells are cultured in modular units, the timing to start the experiments can be controlled by accounting for different maturation times of different tissues or organoids. Additionally, the chip device can be disassembled at the end of the experiment to retrieve the sample for further experiments or analyses.

By employing this platform, we demonstrate the application of this Organ-on-a-Chip platform with a BBB-brain model. We first developed a BBB-in-a-CUBE model by co-culturing primary astrocytes and pericytes in a basement membrane hydrogel (Matrigel) with iPSC-derived BMECs seeded on the top surface of the Matrigel, and confirmed the expression of important tight junction and transporter proteins by immunofluorescence staining and RT-qPCR. We also validated the function of the BBB by measuring the trans-endothelial electrical resistance (TEER), as well as conducting permeability experiments with Lucifer Yellow (passive paracellular diffusion) and Rhodamine123 (P-glycoprotein, PGP substrate transport). Finally, as a proof-of-concept, we designed a BBB-brain chip to contain a BBB-in-a-CUBE and a Glioblastoma-in-a-CUBE model to demonstrate, as reported by Tivnan et al.^29^, that the BBB blocking of the PGP substrate drug Vincristine into the brain can be overcome with the addition of PGP inhibitor Reversan. Thus, we show that this platform can be utilized to achieve complex systems by the combination of several small and simple organoid units, without the need for complicated external pump or power sources.

## Results

The design concept for the organ-on-a-chip platform of this study was to reconstruct separately functional tissue units of an organ in a CUBE culture device, before integrating them together in a chip device to replicate complex tissue-tissue interactions. To demonstrate this concept, we first developed a BBB-in-a-CUBE model and confirmed its barrier and transport functions. Following this, we integrated the BBB-in-a-CUBE with a Glioblastoma-in-a-CUBE to convey the potential application of the platform in drug testing research.

### Reconstruction of BBB in a CUBE

**Fig. 2:**
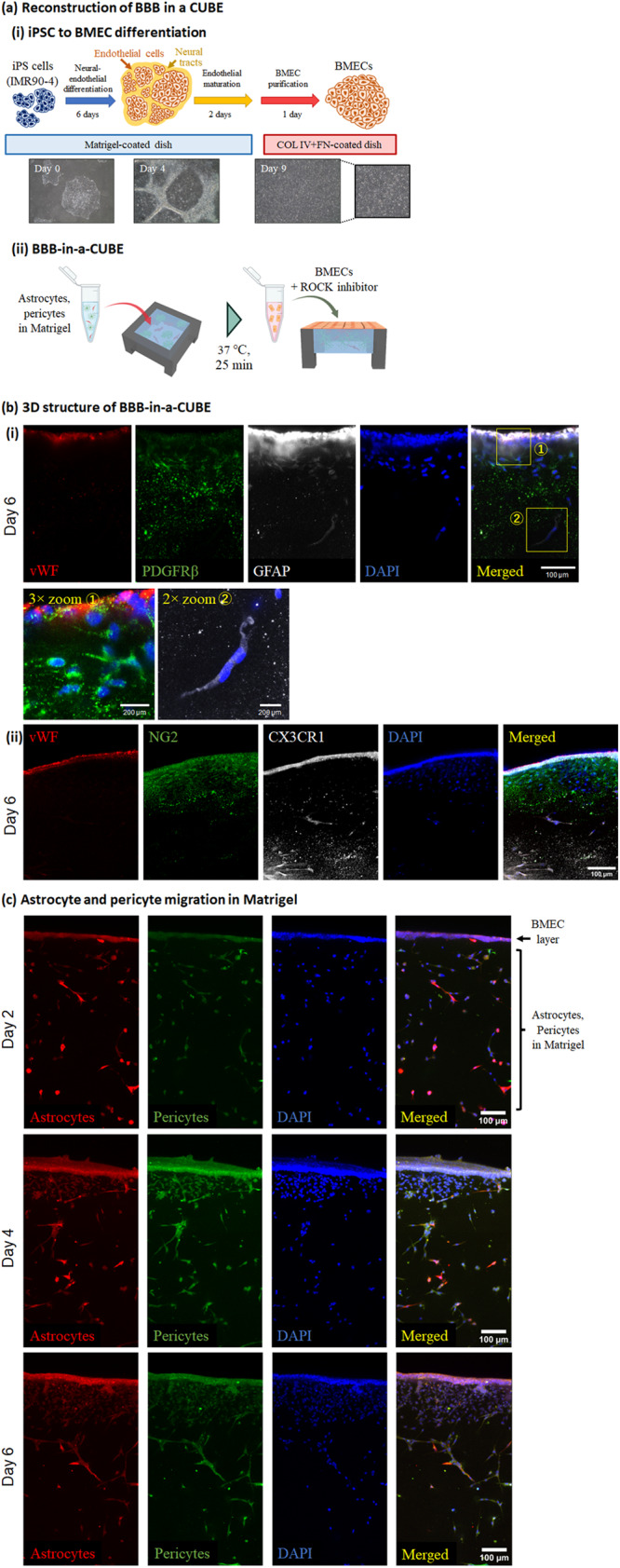
Reconstruction of the blood-brain barrier (BBB) in a CUBE. **a** The structure of the BBB was reconstructed in a CUBE using primary astrocytes and pericytes, and iPSC-derived brain microvascular endothelial cells (BMECs). (i) Differentiation of iPSC to BMEC was based on the protocol by Lippmann et al. (ii) BBB was assembled in the CUBE by first seeding astrocytes and pericytes embedded in Matrigel in the CUBE, then seeding BMECs with ROCK inhibitor Y27632 on the surface of the Matrigel after it has been cured. **b** The 3D structure of the BBB-in-a-CUBE was visualized on Day 6 by labelling BMECs with von Willebrand Factor (vWF), astrocytes with GFAP or CX3CR1, and pericytes with PDGFRβ or NG2, then counterstaining with DAPI. (i) was imaged at 63× magnification with 0.75 zoom factor and (ii) was imaged at 25× magnification with 1.0 zoom factor. **c** BBB-in-a-CUBE on Days 2, 4, and 6 from the side view was visualized by labelling astrocytes with CellLight Tubulin-RFP and pericytes with CellLight Actin-GFP, then counterstaining with DAPI. On Day 2 after seeding, the astrocytes and pericytes were evenly distributed within the Matrigel, but from Day 4 and Day 6, they can be seen to elongate towards and populate the region beneath the BMEC sheet, indicating self-organisation of the BBB structure. Scalebar = 200 µm for enlarged figure and 100 µm for all others.

The function and homoeostasis of the barrier between the blood and the brain is regulated by the synergistic interaction between brain microvascular endothelial cells (BMECs) with the astrocytes and pericytes that surround them^4,30–35^, and most previously developed in vitro BBB models include one or more of these three cell types in their models with or without ECM^5–14,36^. In our model, we reconstruct the 3D structure of the BBB by first embedding primary human astrocytes and pericytes in Matrigel in the CUBE, followed by seeding hiPSC-derived BMECs on the top surface of the Matrigel (Fig. 2a) to replicate the physiological architecture of the BBB and the interactions between astrocytes, pericytes, and BMECs. Imaging of the 3D structure of the BBB-in-a-CUBE with astrocyte markers CX3CR1 or GFAP, pericyte markers NG2 or PDGFRβ, and BMEC marker von Willebrand Factor (vWF) appears to show that the cells arranged into separate layers in the BBB (Fig. 2b), suggesting astrocytes and pericytes are migrating and self-assembling in the Matrigel. Imaging of astrocytes and pericytes fluorescently labelled by transfection showed that over the course of 6 days of culture, astrocytes and pericytes were initially more rounded in shape and evenly distributed in the Matrigel at Day 2 after seeding, but gradually elongated and populated the area under the BMEC layer (Fig. 2c). Quantification of the distribution of astrocytes and pericytes in BBB showed significantly higher number of cells in the region just under the BMEC layer (top) on Day 6 of culture compared to the deeper regions (middle, bottom) of the gel (*p* = 0.011), whereas the cells in Matrigel with only astrocytes and pericytes without BMECs showed evenly distributed cell population in the gel (Supplementary Fig. 1). These results suggest that the presence of BMECs stimulates the astrocytes and pericytes to migrate and elongate towards the BMECs, indicating self-organisation of the cells to form the BBB structure in 3D.

To further clarify the contribution of astrocytes and pericytes to the BBB model, we performed RNA-sequencing analysis on BBB and BMEC only samples (Supplementary Fig. 2). GO analysis of the genes in clusters showing higher expression levels with increasing culture time and clusters where gene expression in BBB was higher than in BMEC only revealed many genes related to cellular migration as well as ECM and vascular remodelling, indicating astrocytes and pericytes are involved in modulating the BBB. Additionally, insulin-like growth factor (IGF) binding protein, which is an important transport system utilized by drug development researchers to deliver biologics to the brain^37–39^, was also more highly expressed in BBB compared to BMEC only, suggesting that astrocytes and pericytes may be required for the expression of some transporter proteins in the BBB.

There is currently no consensus on the optimal seeding density or ratio of the three cell types. Pericyte to BMEC ratio have been reported to be 1:1–1:3 with astrocytes covering 99% of the brain basement membrane^31,40^, and varying ratios of astrocytes:pericytes:BMECs have been used in different in vitro BBB models, including 1:1:10 by Nakagawa et al. 1:1:1 by Harding et al. and 5:1:1 by Al Ahmad et al. and Stone et al.^5,6,41,42^. For our model, we decided on a 1:1 ratio of astrocyte to pericyte, with a seeding density of 1 × 10^4^ cells of each per CUBE as this was the density that we found did not cause shrinkage of the Matrigel over 6 days of culture. BMECs were seeded at 5.5 × 10^4^ cells per CUBE as this was the number of cells required to form a monolayer of cells that covers the entire Matrigel surface and that does not contract and detach from the CUBE after 6 days of culture. Nevertheless, further investigation into the most physiologically relevant composition of the BBB by comparison with in vivo models would be required to properly determine the ideal combination of cell types.

Matrigel was selected as the hydrogel scaffold for the cellular components of the BBB to be grown on as it contains many of the basement membrane components of native BBB such as collagen IV, laminin, nidogen, and perlecan^43,44^. However, the actual composition of Matrigel is not very well-defined, and there are often reported batch-to-batch inconsistencies with Matrigel supply^45^. Additionally, though the thickness of the in vivo basement membrane is about 30–100 nm^44,46^, the thickness of the Matrigel in the BBB-in-a-CUBE is about 1.6 mm, necessitated because of the volume required for the gel to be able to adhere on the CUBE frame by surface tension. However, based on the Day 2 to Day 6 imaging evidence of astrocytes and pericytes migrating towards the surface of the Matrigel closer to the BMECs, it appears that even in a thick Matrigel, the cells can self-organise to form the actual BBB at the surface of the gel. Nevertheless, with the biomaterials field continually evolving to develop alternative biomimetic materials, it may be prudent in the future to consider alternatives such as chemically defined synthetic hydrogels^45^ or an ultra-thin membrane with ECM hydrogel recently developed^47^, as well as to identify the most appropriate ECM composition and property suited for the culture of in vitro BBB that resembles the in vivo BBB.

### Tight junctions of BBB-in-a-CUBE

**Fig. 3:**
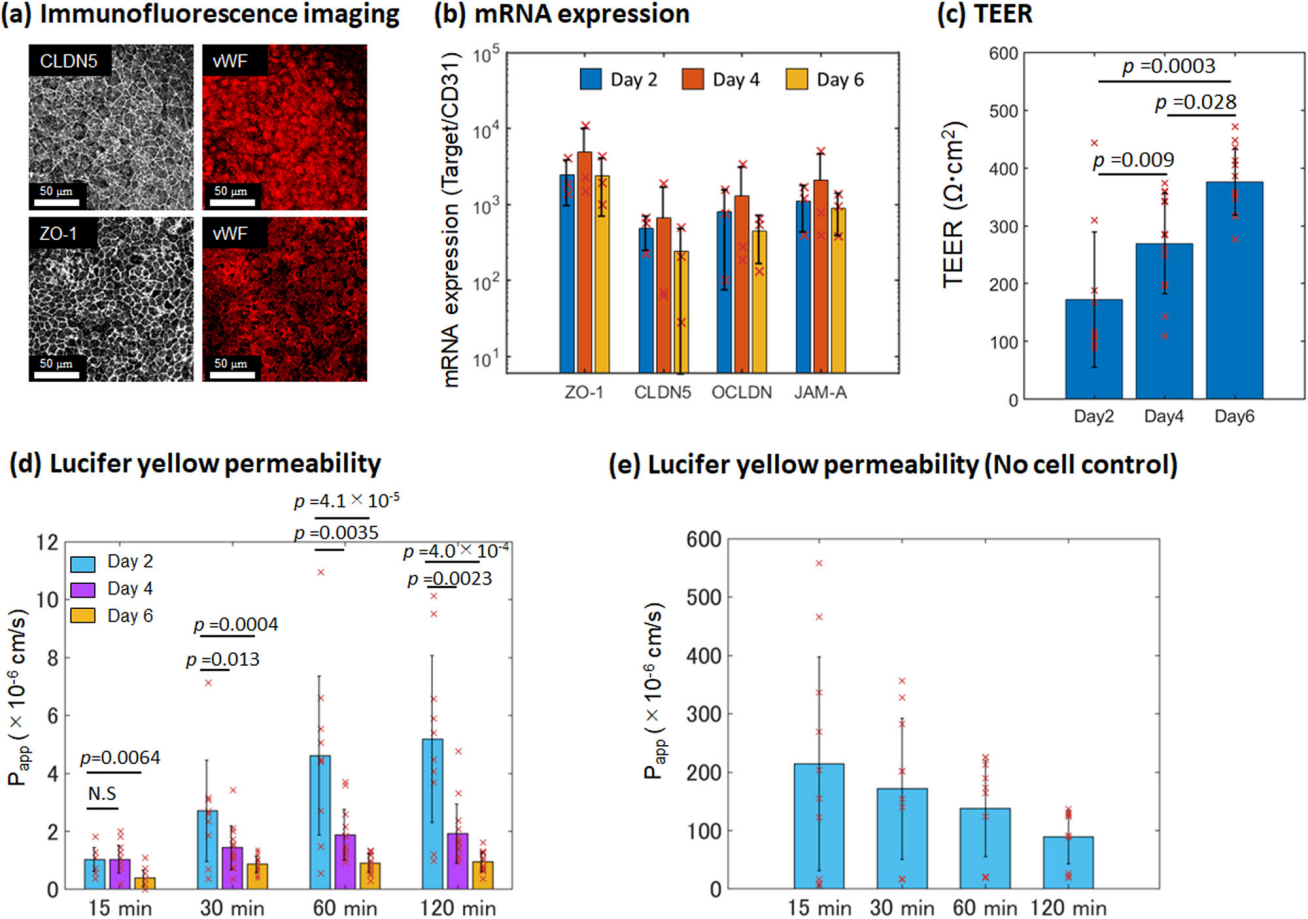
Tight junctions of BBB-in-a-CUBE. **a** Immunofluorescence staining of the tight junction proteins claudin 5 (CLDN) and zonula occludens-1 (ZO-1) with von Willebrand Factor (vWF) marking the BMECs. Scalebar = 50 μm. **b** mRNA expression levels of *ZO-1*, *CLDN5*, occludin (*OCLN*), and junctional adhesion molecule (*JAM-A*) by RT-qPCR. Expression levels were normalized to that of *CD31*. Blue = Day 2, Orange = Day 4, and Yellow = Day 6. **c** Measurement of trans-endothelial electrical resistance (TEER) as a representation of the strength of BBB tight junction showed increasing TEER values over 6 days of BBB-in-a-CUBE culture, indicating stronger barrier formation. **d** The apparent permeability (P_app_) of the BBB-in-a-CUBE to small hydrophilic molecule Lucifer Yellow (LY) decreased over the course of 6 days of culture, confirming the strengthening of tight junction function. Blue = Day 2, Purple = Day 4, and Yellow = Day 6. **e** The much higher P_app_ values obtained when the LY permeability experiment was performed using Matrigel without any cells confirmed that the low P_app_ values were not due to accumulation of LY in Matrigel. Data were collected from 3 independent experiments with 6 technical replicate each for TEER and LY permeability with BBB-in-a-CUBE, and from 1 independent experiment with 10 technical replicates for LY permeability without cells. Bar graph shows average, error bars show standard deviation, and *p* value was calculated by Kolmogorov-Smirnov (KS) test.

The tight junctions of the BBB act as a physical barrier in blocking the passive diffusion of substances from the blood to the brain^48,49^. Immunofluorescence staining show that the BBB-in-a-CUBE expresses claudin-5 (CLDN5) and zonula occludens-1 (ZO-1) (Fig. 3a), and qPCR confirmed the mRNA expressions of *CLDN5*, *ZO-1*, occludin (*OCLN*), and junctional adhesion molecule (*JAM-A*) (Fig. 3b).

TEER measurement is a commonly used method to assess the integrity of endothelial barrier to passive ionic diffusion^50^. The TEER values measured for BBB-in-a-CUBE were 202 ± 110 Ω cm^2^ on Day 2, 299 ± 84 Ω cm^2^ on Day 4, and 405 ± 55 Ω cm^2^ on Day 6 (Fig. 3c), showing increasing TEER as the cells are cultured for longer. Interestingly, BMECs cultured in Matrigel only without astrocytes and pericytes had higher TEER values than BBB initially on Day 2, but did not change much over the culture period (Supplementary Fig. 3a). Furthermore, there was no significant difference in TEER at Day 6 between BMEC only and BBB models, which suggests that the astrocytes and pericytes may not be contributing directly to the strengthening of the barrier in the BBB model in terms of tight junction. These results are also much lower than the estimated 8000 Ω cm^2^ reported in vivo^51^, and are also in contrast to other BBB models using iPSC-derived BMEC where TEER values over 1000 Ω cm^2^ are typically reported, although these tend to peak at about day 1 or 2 before gradually decreasing^7,47,52–54^. To confirm that the difference in our TEER values compared to that in the original differentiation protocol stem from differences in the BBB culture system and not due to differences in differentiation protocol, we performed TEER measurement of our BMECs in the Transwell system. Our Transwell measured TEER showed a peak of 904 ± 477 Ω cm^2^ (Supplementary Fig. 3b). Considering differences in insert size, where TEER measured in 6.5 mm inserts was reported by Vigh et al. ^55^ to be two times lower than the 12 mm inserts used in the protocol by Lippmann et al., our BMECs have comparable TEER to the Lippmann protocol when cultured in Transwells.

The movement of small hydrophilic molecules such as Lucifer Yellow (LY) across the BBB is also often used as an indicator of barrier integrity to passive diffusion of non-electrolytes^50,56^. The LY apparent permeability (P_app_) of BBB-in-a-CUBE was 1 ~ 5 × 10^−6 ^cm/s at Day 2, 1 ~ 2 × 10^−6 ^cm/s at Day 4, and <1 × 10^−6 ^cm/s at Day 6 (Fig. 3d). Inversely correlating to the increasing TEER from day 2 to day 6 of culture, the P_app_ of BBB-in-a-CUBE to LY decreases and also shows lower variability with longer culture, indicating strengthening of barrier function. As barrier permeability of about 1 × 10^−6 ^cm/s are typically used for drug permeability assessments^52,57^, BBB-in-a-CUBE at Day 6 were used for subsequent studies. To confirm that the low permeability results were not due to accumulation of LY in the Matrigel, LY permeability in Matrigel only without cells was also assessed and showed much higher permeability than BBB-in-a-CUBE samples (Fig. 3e). The decreasing LY permeability from 15 min to 120 min in samples without cells may be due to the negatively charged LY becoming trapped in the gel^58,59^, but the amount of LY build-up required for this effect is much higher than the amount that passes through the BBB, so we can consider that the low permeability of BBB-in-a-CUBE was a result of BBB function and not because of LY accumulation in Matrigel.

### Transporters of BBB-in-a-CUBE

**Fig. 4:**
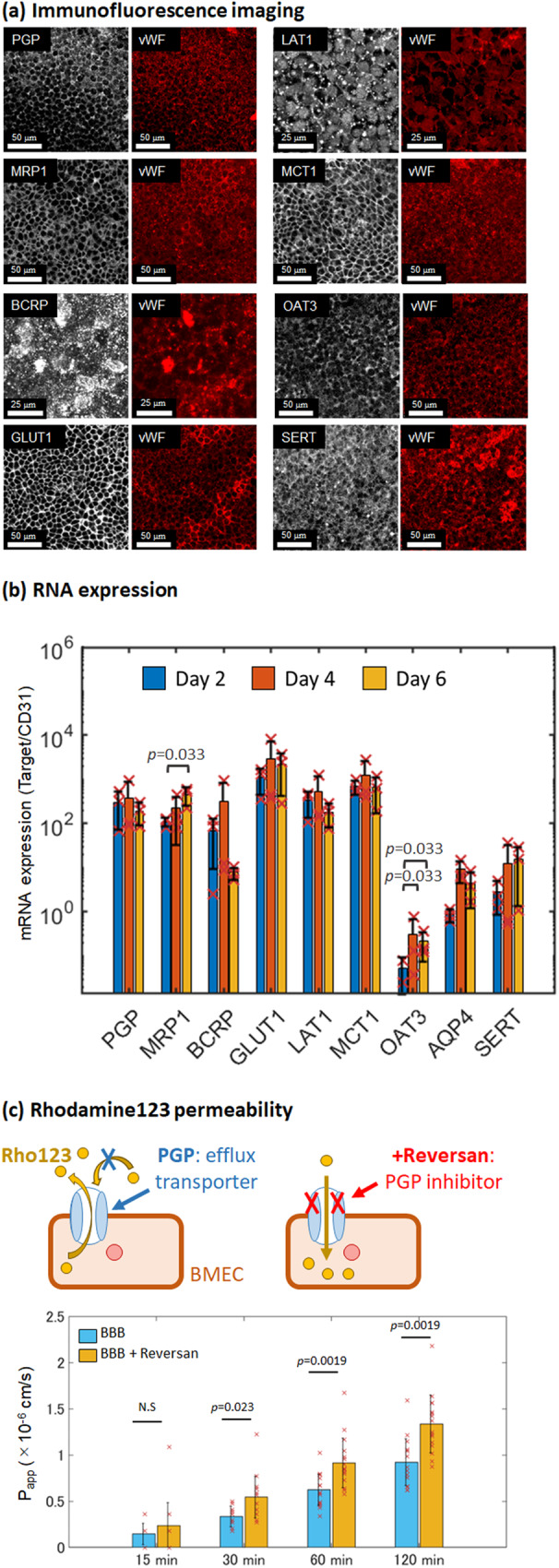
Transporters of BBB-in-a-CUBE. **a** Immunofluorescence staining of the transporters multidrug resistance protein 1 (MDR1/PGP/ABCB1), multidrug resistance-associated protein 1 (MRP1/ABCC1), breast cancer resistance protein (BCRP/ABCG2), glucose transporter 1 (GLUT1/SLC2A1), large amino acid transporter 1 (LAT1/SLC7A5), monocarboxylate transporter (MCT1/SLC16A1), organic anion transporter (OAT3/SLC22A8), and serotonin (SERT) with von Willebrand Factor (vWF) marking the BMECs. Optical zoom = 2 for BCRP and LAT1, and 1 for others; Scalebar = 25 μm for BCRP and LAT1, and 50 μm for the others. **b** RT-qPCR confirmed the mRNA expressions of *MDR1*, *MRP1*, *BCRP*, *GLUT1*, *LAT1*, *MCT1*, *OAT3*, and aquaporin 4 (*AQP4*). Expression levels were normalized to that of *CD31*. Blue = Day 2, Orange = Day 4, and Yellow = Day 6. **c** The apparent permeability (P_app_) of the BBB-in-a-CUBE to the PGP substrate Rhodamine123 (Rho123) increased with the addition of the PGP inhibitor Reversan, indicating that BBB-in-a-CUBE expresses functioning PGP transporters. Blue = BBB without Reversan and Yellow = BBB with Reversan. For Rho123 permeability with BBB-in-a-CUBE, data was collected from 3 independent experiment with 6 technical replicates for each experiment. Bar graph shows average, error bars show standard deviation, and *p* value was calculated by Kolmogorov-Smirnov (KS) test.

The transporters of the BBB mediate the movement of essential nutrients from the blood to the brain, as well as the efflux of toxic or unwanted substances from the brain into the bloodstream to be eliminated^48,49^. Immunofluorescence imaging with scanning from the top BMEC side shows that the BBB-in-a-CUBE expresses multidrug resistance protein 1, also known as p-glycoprotein (MDR1/PGP/ABCB1), multidrug resistance-associated protein 1 (MRP1/ABCC1), breast cancer resistance protein (BCRP/ABCG2), glucose transporter 1 (GLUT1/SLC2A1), large amino acid transporter 1 (LAT1/SLC7A5), monocarboxylate transporter (MCT1/SLC16A1), organic anion transporter (OAT3/SLC22A8), and serotonin (SERT) (Fig. 4a), and qPCR confirmed the mRNA expressions of *PGP*, *MRP1*, *BCRP*, *GLUT1*, *LAT1*, *MCT1*, *OAT3*, and aquaporin 4 (*AQP4*) (Fig. 4b).

The localization of transporters in a cell is also important for its proper functioning. To examine the localization of transporters in BBB-in-a-CUBE, the sample was removed from the CUBE frame and embedded in 1.5% agarose to image the cells from the side view. Immunofluorescence staining results of the samples show that PGP, BCRP, MRP1, and OAT3, which are efflux transporters, had higher expressions on the luminal (blood) side, while GLUT1, MCT1, SERT, and LAT1 were expressed on both luminal and abluminal (brain) sides of the BMECs (Supplementary Fig. 4). Quantification of the intensity of efflux transporter markers confirmed higher expression on the luminal side of BMEC (Supplementary Fig. 5), showing agreement with literature reporting luminal localisation of efflux transporters^56,60–62^.

The permeability of Rhodamine123 (Rho123), a PGP substrate, across the BBB barrier is oftentimes used as an indicator of PGP transporter function^5,7,47,63^. The common method is to measure the efflux ratio of Rho123, which is the ratio of basal-apical flux to apical-basal flux. However, due to the thickness of the Matrigel and potential accumulation of Rho123 in the Matrigel, the efflux ratio may not be representative of the PGP transporter function. Instead, we used the method comparing the permeability of Rho123 with and without the PGP inhibitor Reversan. The Rho123 P_app_ of BBB-in-a-CUBE increased with the addition of Reversan (Fig. 4c), indicating the presence and function of PGP. It should be noted, however, that Reversan is also an inhibitor for MRP1^29,64^ and Rho123 has been reported to be a substrate for organic anion transporting polypeptide (OATP) and organic cation transporters (OCT) transporters^65,66^. Nevertheless, the significant difference between P_app_ of Rho123 with and without Reversan (*p* = 0.0019 ~ 0.023) show that the BBB-in-a-CUBE possesses functioning transporters. More specific investigations beyond the scope of this study, given the large number of transporters that exist, would be required to ascertain whether certain transporters are expressed and functioning in BBB-in-a-CUBE.

### BBB-Glioblastoma interaction

**Fig. 5:**
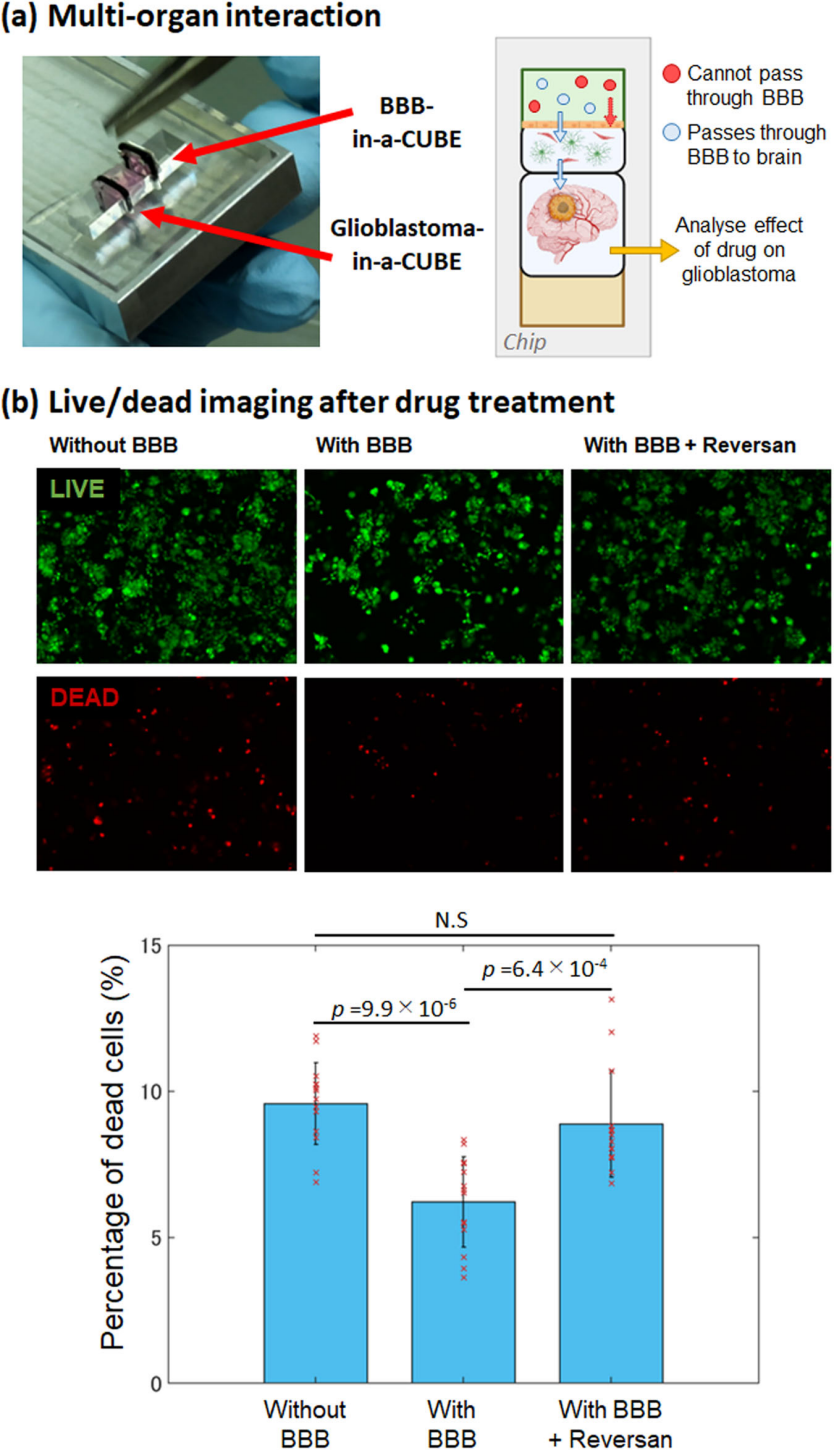
BBB-Glioblastoma interaction. **a** Conceptual diagram of how multi-organ interaction can be achieved by integrating Tissue-in-a-CUBEs together in a chip device. A BBB-in-a-CUBE and a Glioblastoma-in-a-CUBE are positioned together in a chip with their respective growth media in the respective media chambers. Drugs to model the treatment of glioblastoma are then added to the BBB side to determine if they can pass through the BBB, and their effects on the glioblastoma. **b** The proof-of-concept was performed using T98G glioblastoma cells with vincristine, a PGP substrate chemotherapy drug used to treat glioblastoma, as the test drug. The percentage of dead cells was higher when there was no BBB and when the PGP transporter was inhibited with Reversan, compared to when a BBB was present, showing that the drug does not pass through the BBB easily but can be permitted through when the transporter function is inhibited. The results also demonstrated that the effects of the drug on the glioblastoma can be determined by retrieving and analysing the Glioblastoma-in-a-CUBE post experiment. Data was collected from 3 independent experiments with 4–5 technical replicates for each experiment for live/dead imaging in each condition. Bar graph shows average, error bars show standard deviation, and *p* value was calculated by Kolmogorov-Smirnov (KS) test.

To demonstrate the application of BBB-in-a-CUBE in drug testing, we designed a chip to contain a BBB-in-a-CUBE and a Glioblastoma-in-a-CUBE as a brain cancer model to test the permeability and effect of the chemotherapy drug Vincristine, based on the study of Tivnan et al. ^29^ that reported that inhibition of PGP and MRP1 transporters by Reversan increased the effect of vincristine, a PGP and MRP1 substrate^5,7,60^, on glioblastoma cell death. At the appropriate timing to start the drug testing experiment, the two CUBEs were assembled side-by-side in the chip, and vincristine drug added to the BBB side of the assembly (Fig. 5a). As a control, a CUBE containing only Matrigel without the cellular components of the BBB was used in place of the BBB-in-a-CUBE. After 4 hrs, the Glioblastoma-in-a-CUBE was retrieved from the chip and cell death confirmed by live/dead staining. Without the presence of BBB, the percentage of dead T98G glioblastoma cells in Glioblastoma-in-a-CUBE was 9.6%, whereas with BBB-in-a-CUBE, only 6.1% of cells were dead. When Reversan was added with vincristine, the percentage of dead increased to 8.9% (Fig. 5b). The barrier function towards small water-soluble molecules and PGP substrates had been confirmed, respectively, with the LY and Rho123 (~400 Da) permeability tests above. Hence, the significant increase in dead cells when PGP transporter was inhibited (*p* = 6.4 × 10^−4^), which was about the same amount as when there was no BBB (*p* = 9.9 × 10^−6^), must be due to the movement of vincristine through the disruption of PGP transporter function. These results were in agreement with the report that the inhibition of PGP and MRP1 transport allowed vincristine to pass through the BBB and into the brain where it could exert its effect on the brain cancer. Additionally, the multi-tissue platform developed here requires no pump systems or external power source, thus eliminating the complicated setup often associated with microfluidic devices.

## Discussion

Besides its potential application in testing the barrier permeability of a drug and its effect on the target organ, we also envision usage of this platform to study the interaction of different tissues in healthy and diseased states. For example, proper functioning of the BBB is necessary to maintain a healthy brain by regulating homoeostasis and clearing substances harmful from the brain. However, when the BBB becomes disrupted in diseases such as in neurodegenerative diseases, these harmful substances build up in the brain and further disrupts the BBB, causing the disease to worsen progressively^3,15,67^. By culturing the BBB together with a brain organoid developed from iPSCs of a diseased patient in this multi-organ platform, it would be possible to investigate the interplay between the diseased brain and BBB, and vice versa.

The advantage of this platform is that the tissue samples can be cultured separately as individual components before being integrated into a chip device for experiments; this is particularly relevant for tissues that require long culture times. The differentiation of stem cells to organoids typically requires weeks to months of culture for the organoids form, and not all organoids mature successfully. By culturing organoids in the CUBE device, only organoids that are deemed to have matured successfully can be selected to be incorporated in the multi-organ chip at the appropriate timing, thereby reducing wastage compared to if the organoid had been cultured in the chip from the beginning. Furthermore, the modularity of the tissue samples means that they can be easily retrieved later on for further experiments or post-experiment analyses, increasing the usability of each sample.

When co-culturing organoids that have varying differentiation media requirements, the typical approaches to satisfy the needs of both organoids are to mix both types of media, or to apply a constant flow of the different media in separate channels using a microfluidic device. In the setup of this platform, an O-ring is attached to the CUBE to minimise the leakage of medium from one chamber to the other through the gaps between the CUBE and the PDMS chip. Hence, when two organoids with differing differentiation media are integrated together in the chip, they can continue to be cultured with their respective media in the separate media chambers, without the need for complicated flow setups in microfluidic devices with pumps of syringes.

One of the disadvantages of this platform in its current form is the low throughput due to the design of the chip - the commercially available clamp holder required to make the device water-tight and prevent leakage around the CUBE during drug testing could only allow us to fit two sets of test chambers in the device. However, we anticipate increasing the number of test chambers with future customisation and improvement of clamp holder and chip designs to enable higher throughput.

Despite the many advantages of PDMS including biocompatibility, permeability to most gases, optical clarity, and simple fabrication method, the use of hydrophobic PDMS as a material component of the CUBE and chip may also be a cause of concern as proteins and small molecules have been known to be absorbed on the surface of PDMS^68–70^. Nevertheless, several methods have been reported to overcome this issue, including adding poly(ethylene glycol) (PEG) to PDMS to increase its hydrophilicity^71^ or applying Teflon, 2-methacryloyloxyethyl phosphorylcholine (MPC) polymer or paraffin wax as a coating^72–74^. Furthermore, the CUBE and chip can also be fabricated using acrylic, which although reduces slightly the optical clarity of the devices, does not absorb proteins on its surface.

BMECs tend to form tubular network structures instead of monolayers when seeded on Matrigel-coated dishes with the addition of vascular endothelial growth factor (VEGF); tube formation did not occur on Matrigel-coated dishes without the VEGF^7,75,76^. Yet, in our BBB-in-a-CUBE model, BMECs formed tube-like structures even without the addition of VEGF. This is likely because of the softer substrate stiffness of a thick Matrigel in the CUBE leading to tube formation compared to uniformly distributed cells on stiffer dish substrate, as substrate stiffness has been reported to affect tubular network formation in endothelial cells^77^. However, we found that the addition of the selective Rho-associated protein kinase (ROCK) inhibitor Y-27632, which has been shown to enhance endothelial cell adhesion to substrates and promote wound healing^78,79^, enabled the seeding of BMECs as a monolayer on Matrigel. RhoA/ROCK inhibition has been reported to suppress VEGF-induced endothelial migration and tube formation in BMEC but did not affect basal endothelial cell migration^80–82^, which may be the reason that BMECs could adhere to Matrigel in the CUBE as a monolayer instead of forming tubular networks. Nevertheless, as RhoA/ROCK activity is involved in many endothelial and vascular processes such as migration, proliferation, inflammation, and maintenance of barrier integrity, Y-27632 was only included in the first day of culture.

Although primary or immortalized cell lines of animal origin were commonly used in many previous BBB models due to their easier availability, high TEER and low permeability values compared to human cells^50^, iPSC-derived cells, particularly iPSC-derived BMECs based on the work by Lippmann et al.^7,53^, are currently the preferred cell source due to their essentially unlimited supply obtainable by simple differentiation protocols and negation of human-animal species differences, whilst still having high TEER and low permeability.

Some studies have recently claimed that the BMECs obtained based on the protocol established by Lippmann et al. also possess an epithelial profile in addition to endothelial profile^83–85^. Nevertheless, Lippmann et al. asserted in a commentary that the iPSC-derived BMECs still possess vascular profile and function despite also having an epithelial profile, and care should be taken in choosing BMEC derivation protocols depending on the experimental purposes^86^. As the main concept of this study was to develop a modular CUBE platform to replicate tissue–tissue interactions, the choice of BMECs (or other cell types) can be customised depending on the user’s requirements. Interestingly, however, when we extracted endothelial and epithelial markers described in the article by Lu et al. ^83^ from the RNA-seq data, we found that in our BBB model, expression of endothelial markers such as *vWF*, *ETS1*, *FLI1*, and *ERG* increased over the 6-day culture period while the epithelial markers such as *FREM2*, *EPCAM*, *TRPV6*, and *KRT8* decreased (Supplementary Fig. 6). This suggests that longer culture in the appropriate environment (co-culture with astrocytes and pericytes in a basement membrane hydrogel) may play a role in guiding the BMECs to acquire characteristics more representative of the in vivo conditions, although further investigation into this is beyond the scope of this paper. Thus, the choice of differentiation and culture protocol should be chosen depending on the desired properties of the BBB that is the priority, whether that be the highest TEER value, expression and function of certain transporter proteins, or having fully endothelial transcriptomic profile.

A possible reason for the TEER difference is the substrate on which the cells are seeded. Prevailing TEER measurement is based on the equation; TEER = (R – R_*blank*_) × SA. Subtracting the electrical resistance force in the blank condition from the measured sample resistance removes the electric noise in the experimental system (e.g. Transwell culture, microfluidics, CUBE) but does not fully take into account pore size differences. While TEER calculations are based on cell seeding area, in the other studies, BMECs were seeded on a porous synthetic membrane, where only 10–15% of the seeding area is actually permeable to ions. On the other hand, BMECs in BBB-in-a-CUBE were seeded on Matrigel only where the whole surface area is permeable to ion movement. In fact, there are increasingly concerns being raised about methods of TEER measurements and calculations, as it is difficult to make comparisons between TEER values reported in different papers due to the results being easily affected by many parameters including substrate porosity, surface area, and stiffness, as well as measurement equipment^55,87–89^. The higher resistance of synthetic membranes to electrical current may also explain the higher TEER values compared to hydrogel only substrate. Furthermore, it has been reported that BMECs cultured on collagen gels in Transwells (~840 Ω cm^2^) showed lower TEER than those cultured directly on Transwells (~5500 Ω cm^2^)^90^, indicating that the composition and stiffness of the substrate may also contribute phenotypic changes to the cells and may require further investigations in future work, as discussed in the previous section. Regardless of issues that may arise from methods to determine TEER, since establishing a new TEER measurement method is not within the scope of this paper, we utilized the current TEER method as a measure of tight junction barrier function over the BBB culture period. Although the TEER values measured in this study are low compared to those reported in vivo or other iPSC-derived BMEC papers, the low permeability to LY shows that BBB-in-a-CUBE possesses sufficient barrier function. This claim can be backed up by evidence that shows that even though TEER value becomes greater than 150 Ω cm^2^, the supposed increase in barrier tightness was not reflected in further decreases in permeability measurements^91^.

Although astrocytes and pericytes have been included in several BBB models and resulted in tighter barrier function, the astrocytes and pericytes were often in separate compartments of the Transwell and not interacting directly with one another or with the basement membrane^5,7,53^. In the BBB-in-a-CUBE model, the close interaction of the three cell types with the basement membrane may have allowed the cells to mature and self-organise to form a more stable BBB. On the one hand, the increase in TEER over 6 days of culture in the BBB model may be due to the physical presence of astrocytes and pericytes that have migrated close to the BMEC layer, thus increasing electrical resistance of the tissue. Given that there was no significant difference in the TEER values at Day 6 between BBB and BMEC only models (Supplementary Fig. 3a), and the expressions of tight junction and transporter markers in BBB remained stable over the culture period (Figs. 3b and 4b), it suggests that astrocytes and pericytes do not contribute directly to the tightness of the barrier function, at least in terms of the parameters in these particular experiments. On the other hand, the astrocytes and pericytes may also be contributing other biological functions important to BBB function, as RNA-seq analysis revealed increases in CD31 and CLDN5 expressions in BBB compared to BMEC only (Supplementary Fig. 6) as well as in IGF transporter and ECM remodelling related expressions (Supplementary Fig. 2). Thus, the choice of BMEC only or BBB model to use depends on the purpose of the experiment to be conducted and whether astrocyte and pericyte interaction with BMEC is an important factor to answer the research question. This may also be an interesting avenue to pursue in future work investigating the potential synergistic effects of BBB components in health or disruptive effects in disease.

With differentiation protocols for iPSC-derived astrocytes and pericytes also increasingly being developed^92–96^, ideally fully iPSC-derived models, particularly patient-derived models, could be developed to study human diseases in vitro and provide for personalized medicine in the future. However, for this study, primary astrocytes and pericytes were used because the differentiation protocols for iPSC-derived astrocytes and pericytes typically require weeks to months of culture, while commercially available iPSC astrocytes and pericytes cannot be passaged and expanded, rendering both options unsuitable for the purposes of this study in terms of cost.

In this study, we established an Organ-on-a-Chip platform utilizing our previously developed CUBE culture device to culture modular tissues or organs that can be assembled in a chip to recapitulate multi-organ interactions. Compared to traditional Transwell systems, the CUBE and chip platform enables the reconstruction of complex 3D tissues or organoids in the CUBE device and the integration of multiple CUBEs to achieve even higher complexity. It is anticipated that the platform can be widely adopted by biology-based researchers and further developed into complex in vitro model systems that can reduce the reliance of animal models in both basic research and drug testing.

## Methods

### CUBE and chip fabrication

CUBEs, moulds to fabricate the chip, and chip base holders were designed using Rhinoceros 3D software (Robert McNeel & Associates) and ordered from a machining company (Proto Labs Japan). All CUBEs, chip moulds, and chip base holders were made of aluminium. Design dimensions and methods to fabricate PDMS are shown in Supplementary Fig. 7.

The original CUBE design was modified with a thicker frame on the top side of the CUBE to allow the attachment of a nitrile O-ring (AS ONE, 62-3049-63) to the CUBE, which was necessary to ensure a tight seal between CUBE and chip^28^. Two types of CUBEs were used in this study: (i) for BBB-in-a-CUBE, the bottom half of the CUBE was removed to reduce the amount of Matrigel used, and (ii) for Glioblastoma-in-a-CUBE, the sidewalls of the CUBE were covered with PDMS (Supplementary Fig. 7a). PDMS (Silpot 184, Dow Toray, 04133124) was prepared by mixing the elastomer base with the curing reagent at a 10:1 ratio. To make PDMS sidewall with a thickness equivalent to that of the CUBE frame (0.75 mm), ~2.8 g of the mixture was spread out in a 100 mm dish and degassed to remove air bubbles, before placing CUBE frames on the dish. The PDMS was degassed again and baked at 85 °C for 20–30 min. After curing, excess PDMS was trimmed from the frames with a scalpel, and the process repeated for the remaining three adjacent sides of the CUBE, leaving the top and bottom surfaces open (Supplementary Fig. 7b).

Two types of chips were used in this study: (i) for TEER measurements and permeability tests, the chip moulds were designed to hold the BBB-in-a-CUBE with two media chambers at the top and bottom sides of the CUBE, and (ii) for BBB-brain interaction experiments, the chip moulds were designed to fit BBB-in-a-CUBE and Glioblastoma-in-a-CUBE together, with media chambers on the two ends of both CUBEs (Supplementary Fig. 7c). Each chip comprises a base component which holds the CUBEs and media compartments, and a lid component with access ports to seal the chip. PDMS chips were made by pouring uncured PDMS into the mould, degassing to remove air bubbles, then baked at 85 °C for about 1 h. After curing, the PDMS chips were pried out of the mould (Supplementary Fig. 7d).

Prior to use with cell culture, CUBEs and chips were washed with ultrasonication once in MilliQ water and twice in isopropanol (IPA), for 10 min each wash, then dried in the oven for 2 h. O-ring was washed as follows: soak in acetone for ~4 h, discard acetone and replace with fresh acetone to soak overnight, discard acetone and ultrasonicate with IPA for 10 min, discard IPA and ultrasonicate with autoclaved MilliQ water for 10 min, then spread O-rings out in a dish and leave to dry overnight. To assemble the CUBE and chip setup, a double-sided adhesive film (NSD-100, NIPPA) was attached onto the lid component of the chip and holes punched in the access ports to allow media to be added to the chip later. The chip base was placed in a holder to prevent it from overexpanding when it is sealed. Then, the CUBEs were placed in the base component, and the adhesive lid placed on top of the base to seal the chip. The sealed chip was then placed in a clamp holder to ensure a tight seal of the PDMS chip, and media can be added to the media chambers via the access ports. The clamp holder was purchased from Micronit (Fluidic Connect PRO Chipholder Frame; FCPROCH) with an attachment customized to fit our chip design ordered from a microfabrication company (Icomes Lab, Japan) (Supplementary Fig. 7e, Supplementary Movie 1).

### Cell culture

Primary normal human astrocytes (NHA; Lonza, CC2565) were cultured in astrocyte growth medium (AGM Bulletkit; Lonza, CC3186) supplemented with 0.7% Penicilin-Streptomycin (PS; Gibco, 15140122). Primary human brain vascular pericytes (HBVP; ScienCell, 1200) and glioblastoma multiforme cell line T98G (RIKEN Cell Bank, RCB1954) were cultured in DMEM/F12 with GlutaMAX supplement (Gibco, 10565018) supplemented with 10% foetal bovine serum (FBS; Cytiva, SH30396.03) and 1% PS. NHA, HBVP, and T98G were dissociated using 0.25% Trypsin-EDTA (Gibco, 25200-056) and trypsin neutralizing solution (TNS; Kurabo, HK3220). NHA and HBVP were used within passages p4–7, and T98G used within p7–13 in experiments. IMR90-4 iPSCs (WiCell, WB65317; karyotyped as normal by supplier) between passages p35–45 were maintained on Matrigel-coated dishes in mTeSR Plus medium (STEMCELL Technologies, 100-0276) and dissociated using ReLeSR (STEMCELL Technologies, 05872). For Matrigel coating, hESC-qualified Matrigel (Corning, 356231) was diluted 1:100 in DMEM/F12, and 1 mL of the diluted Matrigel was used to coat one 35 mm culture dish. The dish was incubated at room temperature for one hour, and rinsed once with 1× DPBS (Gibco, 14190) before use. For use in differentiation, IMR90-4 were dissociated using Accutase (Invitrogen, 00-4555-56) and plated with mTeSR Plus with 10 μM Rock inhibitor (Y27632; Nacalai Tesque, 08945-84) for the first 24 hr, then without Y27632 from the next day. When the IMR90-4 reached ~70% confluency, differentiation to BMEC was initiated according to the protocol of Lippmann et al.^7,53^. On Day 0 of differentiation, medium was switched to neural-endothelial differentiation medium which was DMEM/F12 Ham without L-glutamine (Sigma-Aldrich, D6421) supplemented with 20% KnockOut serum replacement (KOSR; Life Technologies, 10828010), 0.1 mM 2-mercaptoethanol (Nacalai Tesque, 21438-82), 1% MEM non-essential amino acid (NEAA; Nacalai Tesque, 06344-56), and 1% GlutaMAX supplement (Gibco, 35050061). After 6 days of neural-endothelial differentiation, the medium was switched to BMEC maturation medium, which comprises human endothelial serum-free medium (hESFM; Gibco, 11111044) with 1% human platelet poor-derived serum (HS; Sigma-Aldrich, P2918), 20 ng/mL basic FGF (bFGF; Fujifilm Wako, 060-04543), and 10 μM all-trans retinoic acid (ATRA; Nacalai Tesque, 36331-44). To coat a dish with collagen IV (COL IV) and fibronectin (FN), 4 mL of a mixture of 50 μg/mL COL IV (Sigma-Aldrich, C7521) and 25 μg/mL FN (Sigma-Aldrich; F2006) in DPBS was used to coat one 100 mm culture dish and incubated at 37 °C for at least 2 h. The excess solution was aspirated and left to dry overnight before use. After 2 days of BMEC maturation, cells were dissociated using Accutase for 20–25 min and re-plated on COLIV + FN-coated dishes in BMEC maturation medium and incubated for 1 h. After 1 h, the cells were washed twice gently with 1× DPBS, then fresh BMEC maturation medium was added, and the cells incubated for another day before use in making BBB. For experiments to visualize the movement of astrocytes and pericytes in the BBB model over the course of the culture period, astrocytes were labelled with CellLight Tubulin-RFP (Invitrogen, C10614) and pericytes with CellLight Actin-GFP (Invitrogen, C10582), overnight prior to making BBB-in-a-CUBE, according to the manufacturer’s protocol.

### BBB-in-a-CUBE

Before seeding cells in the CUBE, an O-ring was attached to the thicker part of the CUBE frame. Astrocytes (0.5 × 10^6^ cells/mL) and pericytes (0.5 × 10^6^ cells/mL) were suspended in Matrigel, and 20 μL of the cell suspension was added to each CUBE, before incubating at 37 °C for 25 min for Matrigel to cure. After Matrigel has cured, 10 μL of BMEC (5.5 × 10^6^ cells/mL suspended in BMEC maturation medium with 10 μM Y27632) was seeded on the top surface of the gel, then incubated at 37 °C for 1 hr for BMEC to attach onto the Matrigel. After BMECs have adhered, the CUBEs were transferred to a 48-well plate containing BMEC maturation medium with 10 μM Y27632. The following day, medium was switched to BBB medium which comprises a 1:1 mix of EC medium (hESFM+1% HS) and AGM. Medium was changed every other day (on Days 3 and 5) by discarding and replacing with fresh half the volume of medium. BBB-in-a-CUBE were used at Days 2, 4, and 6 for TEER measurements and Lucifer Yellow permeability tests, and at Day 6 for Rhodamine123 permeability tests and BBB-brain experiments. BMEC only control was made with the same method as BBB but without the addition of astrocytes and pericytes. A/P only sample was made with the same method as BBB but without the addition of BMEC. A CUBE with 20 μL Matrigel only without cells was used as a No Cell control condition.

### Glioblastoma-in-a-CUBE

Before seeding cells in the CUBE, an O-ring was attached to the thicker part of the CUBE frame. T98G cells were suspended in Matrigel at 1.0 × 10^6^ cells/mL, then added to the CUBE and incubated at 37 °C for 25 min for Matrigel to cure. After curing, the CUBEs were transferred to a 48-well plate containing T98G medium. Glioblastoma-in-a-CUBE were used at Day 3 for BBB-brain experiments.

### Immunofluorescence staining

To fix the cells, BBB-in-a-CUBE samples were washed with DPBS twice, fixed with 4% paraformaldehyde for 20 min, then washed with DPBS for 10 min twice. For immunofluorescence staining, permeabilization was performed by incubating samples in 0.5% Triton X-100 for 20 min, then washing with 100 mM Glycine for 10 min three times. Immunofluorescence buffer (IF buffer) consisted of 0.5% Tween20, 2% Triton X-100, and 10% bovine serum albumin (BSA; Sigma, 126615) in DPBS. Samples were blocked in IF buffer with 10% goat serum (Gibco, 16210064) (IF + G) for 30 min, followed by IF + G with 1% goat anti-mouse IgG (Bethyl Laboratories, A90-116A) for 20 min. Antibodies were prepared according to the dilutions in Table 1 in IF + G with 1% goat anti-mouse IgG. Primary antibody incubation was overnight at 4 °C and secondary antibody incubation was 2 h at RT. After each antibody incubation, samples were washed with IF buffer for 15 min three times. Nuclei were stained with DAPI for 20 min, then washed with DPBS for 5 min three times. For imaging of the 3D structure of BBB, samples were treated with RapiClear clearing solution (SUNJin Lab, RC149001) overnight prior to imaging.

**Table 1:** List of antibodies for immunofluorescence staining

Reagents	Source, Identifier	Dilution
Anti-SLC7A5/LAT1 Rabbit monoclonal	Abcam, ab208776	1:200
Anti-MRP1 Rabbit monoclonal	Abcam, ab23383	1:100
Anti-GLUT1 Rabbit monoclonal	Abcam, ab115730	1:100
Anti-BCRP/ABCG2 Rabbit monoclonal	Abcam, ab207732	1:100
Anti-PGP/MDR1 Rabbit polyclonal	Abcam, ab235954	1:100
Anti-OAT3 Rabbit polyclonal	Abcam, ab247055	1:100
Anti-Serotonin Rabbit polyclonal	Abcam, ab272912	1:500
Anti-MCT1 Rabbit polyclonal	Abcam, ab85021	1:20
Anti-ZO1 Rabbit polyclonal	Abcam, ab216880	1:200
Anti-Claudin5 Rabbit polyclonal	Abcam, ab15106	1:200
Anti-von Willebrand Factor Sheep polyclonal	Abcam, ab111713	1:50
Anti-CX3CR1 Goat polyclonal	R&D Systems, AF5825	2 µg/mL
Anti-GFAP Chicken polyclonal	Abcam, ab4674	1:500
Anti-NG2 Rabbit polyclonal	Abcam, ab129051	1:200
Anti- PDGFRβ Rabbit monoclonal	Abcam, ab32570	1:100
Goat anti-rabbit AF488	Invitrogen, A32731	1:200
Goat anti-rabbit AF647	Invitrogen, A32733	1:200
Goat anti-chicken AF647	Invitrogen, A21449	1:200
Donkey anti-goat AF488	Invitrogen, A11055	1:200
Donkey anti-sheep AF555	Invitrogen, A21436	1:200

### Imaging and Quantification

Samples to be imaged were removed from the CUBE using a 3D-printed jig as described previously^28^ and embedded in 1.5% agarose. When the agarose has cured, samples were rotated onto their sides in the imaging chamber (Ibidi µ-Slide 8 Well, 80826-90) containing DPBS to image the side view of the BBB (Supplementary Fig. 8a). Imaging was performed using a confocal microscope (Leica Microsystems, TCS SP8 Lightning). 25× lens (FLUOTAR VISIR 25×/0.95 water) was used for astrocyte and pericyte migration, 3D structure of BBB, and side-view transporter imaging, and 63× lens (HC PL APO CS2 63×/1.30 GLYC) with Type G immersion liquid (Leica Microsystems, 11513910) was used for top-view tight junction and transporter imaging.

For the quantification of astrocyte and pericyte distribution and migration in Matrigel, a region of 600 µm × 200 µm was cropped 20 µm from the border of the Matrigel to exclude the BMEC layer. The cropped region was then divided into 3 sections of 200 µm × 200 µm and the “Histogram” function of ImageJ used to obtain the highest and lowest count value after adjusting the threshold of the image. The percentage area occupied by cells was calculated by dividing the lowest count value by the total count value (highest + lowest) (Supplementary Fig. 1a). Imaging was performed on 4 technical replicates from 2 independent experiments.

To quantify localisation of efflux transporters (PGP, BCRP, MRP1, and OAT3), an area of an individual cell was cropped and the transporter intensity across the cropped area measured using “Plot Profile” in ImageJ. Each cell image was divided into 3 regions of equal size and the average intensity of each region calculated (Supplementary Fig. 5a). Imaging was performed on 5 technical replicates from 2 independent experiments and 3– 5 cell images were analysed from each replicate.

### Reverse transcription quantitative polymerase chain reaction (RT-qPCR)

Cells were harvested from BBB-in-a-CUBE by detaching the BBB from the CUBE into cold DPBS in a dish, then transferring the BBB to a tube with Cell Recovery Solution (Corning, 354253) for 20 min on ice. Cells were pelleted by centrifugation, and washed with cold DPBS after discarding the supernatant. Centrifugation and DPBS wash steps were repeated before proceeding with RNA extraction. Total RNA was extracted using Total RNA Extraction Miniprep System (Viogene, GR1001) and quantified using Eppendorf BioSpectrometer basic. 8 technical replicates from one independent experiment were pooled together in one tube, and the total RNA was used to prepare the cDNA using SuperScript IV VILO Master Mix with ezDNase Enzyme (Invitrogen, 11766050) according to the manufacturers’ protocols; 3 independent experiments were performed in this study. qPCR was carried out using SYBR Green Realtime PCR Master Mix (Toyobo, QPK-201) according to the manufacturer’s protocol for use with amplification and detection by Analytik Jena qTower^3^: 95 °C for 1 min, and 40 cycles of 95 °C for 15 s, 60 °C for 30 s, and 72 °C for 1 min. Primer sequences are listed in Table 2. Primers were ordered from Eurofins Scientific with sequences obtained from OriGene Technologies. The mRNA expression level was calculated as the fold change 2^-ΔΔCt^, where ΔΔC_t_ is obtained by subtracting ΔC_t_ of the reference gene *CD31* from the ΔC_t_ of the target gene. To identify a suitable reference gene, qPCR was performed with endothelial markers *CD31*, *CD146*, and *eNOS* (Supplementary Fig. 8b), and *CD31* was selected as the most stable based on evaluation by RefFinder (http://blooge.cn/RefFinder/).

**Table 2:** Forward and reverse primer sequences for qPCR

Gene	Forward (5’-3’)	Reverse (5’-3’)
ZO-1	GTCCAGAATCTCGGAAAAGTGCC	CTTTCAGCGCACCATACCAACC
CLDN5	ATGTGGCAGGTGACCGCCTTC	CGAGTCGTACACTTTGCACTGC
OCLN	ATGGCAAAGTGAATGACAAGCGG	CTGTAACGAGGCTGCCTGAAGT
JAM-A	GTGAAGTTGTCCTGTGCCTACTC	ACCAGTTGGCAAGAAGGTCACC
PGP	ACAACCGGCTTCCGCTTGAGAA	ACGCAGTCGAAAATGAAGCGGC
MRP1	CCGTGTACTCCAACGCTGACAT	ATGCTGTGCGTGACCAAGATCC
BCRP	GTTCTCAGCAGCTCTTCGGCTT	TCCTCCAGACACACCACGGATA
GLUT1	TTGCAGGCTTCTCCAACTGGAC	CAGAACCAGGAGCACAGTGAAG
LAT1	GCCACAGAAAGCCTGAGCTTGA	ATGGTGAAGCCGATGCCACACT
MCT1	TTGTTGGTGGCTGCTTGTCAGG	TCATGGTCAGAGCTGGATTCAAG
OAT3	CAACAGCACCAAGGACTCCATTG	CTGTCAGACAGGTCTCCAAGCA
AQP4	GCCATCATTGGAGCAGGAATCC	ACTCAACCAGGAGACCATGACC
CD31	AAGTGGAGTCCAGCCGCATATC	ATGGAGCAGGACAGGTTCAGTC
eNOS	GAAGGCGACAATCCTGTATGGC	TGTTCGAGGGACACCACGTCAT
CD146	ATCGCTGCTGAGTGAACCACAG	CTACTCTCTGCCTCACAGGTCA
TATA	TGTATCCACAGTGAATCTTGGTTG	GGTTCGTGGCTCTCTTATCCTC

### RNA-sequencing and analysis

Total RNA was extracted using as above for BBB days 2, 4, and 6; BMEC only days 2, 4, and 6; A/P only day 6. Library preparation (Stranded RNA-seq library) and sequencing (NextSeq2000, P2, 100 cycles) were outsourced to the Laboratory for Developmental Genome System, RIKEN BDR. To investigate the contribution of co-culturing astrocytes and pericytes with BMEC to BBB maturation, genes with significantly (false discovery rate, FDR < 0.05) higher expression levels in day 6 A/P only compared to day 6 BMEC only were first identified (2966 genes) and excluded to eliminate the effects from just having the presence of astrocytes and pericytes. Then, genes that were consistently higher or lower in day 2, 4, and 6 BBB compared to day 2 BMEC only were extracted (114 genes) to study the synergistic effects of co-culturing BMEC with astrocytes and pericytes over the 6-day culture period. Heatmap was generated using Heatmapper (http://www.heatmapper.ca/) with complete linkage clustering and Pearson distance measurement. GO analysis was performed using g:GOSt functional profiling in g:Profiler (https://biit.cs.ut.ee/gprofiler/gost). To investigate endothelial and epithelial marker expressions in BBB and BMEC only, 12 epithelial markers and 11 endothelial markers from Lu et al. ^83^ were extracted from the whole gene list and a heatmap generated using Heatmapper with single linkage clustering and Kendall’s Tau distance measurement.

### Trans-endothelial electrical resistance (TEER) measurement

Assay buffer comprised 0.01 M HEPES (Gibco, 15630-080), 1 mM sodium pyruvate (Nacalai Tesque, 06977-34), and 0.5% dimethyl sulfoxide (DMSO; Nacalai Tesque, 13445-74) in HBSS buffer (Nacalai Tesque, 09735-75), pH 7.4. To measure TEER, CUBEs were transferred into the TEER chip and assembled as described above. 200 μL of assay buffer warmed to 38 °C was added to each medium chamber and electrical resistance, R was measured using Millicell ERS-2 Volt/Ohmmeter (Merck, MERS00002). All procedures were performed on a 38 °C hotplate. TEER was calculated by subtracting the resistance of the BBB sample (R_BBB_) by that of the No cell control sample (R_blank_) and multiplying by the surface area of the gel (SA = 0.1225 cm^2^). TEER was measured for Days 2, 4, and 6, and the samples were used for Lucifer Yellow permeability tests immediately after TEER measurements. For BBB samples, 2 ~ 6 technical samples were measured in each independent experiment, and 3 independent experiments were performed. For blank samples, 6 samples were measured 3 times each. To confirm that our iPSC-derived BMECs has similar TEER to that in the original differentiation protocol, BMECs were seeded in 6.5 mm polyester membrane Transwell with 0.4 µm pore size (Corning, 3470), cultured in ECM + AGM medium, and TEER measured every day for 4 days. 6 technical replicates from one independent experiment were measured for the Transwell experiment.

### Permeability Test

The setup for permeability tests is the same as for TEER as described above. 200 μL of assay buffer warmed to 38 °C was added to each medium chamber and the samples incubated at 37 °C for 30 min prior to permeability experiment. To start the permeability test, the assay buffer was discarded, and fresh assay buffer added to the basal (astrocyte/pericyte) side of the CUBE while assay buffer with 10 μM Lucifer Yellow (LY; Fujifilm Wako, 125-06281) or Rhodamine123 (Rho123; Fujifilm Wako, 187-01703) was added to the apical (BMEC) side. For Rho123 experiments, samples were incubated with 40 μM PGP inhibitor Reversan (Sigma-Aldrich, SML0173) or an equal volume of DMSO as control for 4 hr prior to permeability experiments. At the designated time points (15, 30, 60, and 120 min), 50 μL of assay buffer was collected from the basal side for fluorometric measurement and replaced with the same volume of fresh buffer. A microplate reader (Molecular Devices, SpectraMax iD3) was used to measure the concentration of LY (Ex. 488 nm; Em. 575 nm) and Rho123 (Ex. 428 nm; Em. 536 nm). Apparent permeability, P_app_ was calculated as follows:

$${P}_{{app}}({cm}/s)=\frac{{Basal}\,{concentration} \times {Basal}\,{volume}}{{SA} \times {Initial}\,{concentration} \times {Time}}$$

6 technical samples were measured in each independent experiment, and 3 independent experiments were performed in this study. Samples with abnormally high concentration at *t* = 15 min (greater than 0.01 μM) were considered to have suffered damage or there was leakage in the chip, and the results were discarded.

### Tissue-tissue interaction

For BBB-Glioblastoma experiments, BBB-in-a-CUBE at Day 6 were incubated with 40 μM Reversan or an equal volume of DMSO as control for 4 hr before being transferred to a chip with Glioblastoma-in-a-CUBE at Day 3, and the chip was assembled as described above. Glioblastoma medium was added to the basal side and BBB medium with 5 μM Vincristine (Tocris, 1257) added to the apical side. Following a 4 hr incubation at 37 °C, the media were discarded and Glioblastoma CUBEs retrieved from the chip. The CUBEs were washed once in live cell imaging solution (LCIS; Invitrogen, A14291DJ), then incubated with 1.5 μM Calcein-AM (Nacalai Tesque, 19177-14) and 1.5 μM propidium iodide (Nacalai Tesque, 19174-31) diluted in LCIS for 20 min at 37 °C. Samples were washed once in LCIS before LIVE/DEAD imaging using a fluorescence microscope with sectioning function (BZX-700, Keyence) equipped with 10× lens (NIKON PlanFluor 10×/0.30). Projection image of 3 slices with 20 µm pitch (40 µm total thickness) was taken from the side closest to the BBB for analysis. The number of live and dead cells were counted using Imaris software (Bitplane, v9.0.2).4–5 technical samples were measured in each independent experiment, and 3 independent experiments were performed in this study.

### Statistics and reproducibility

Number of independent experiment repeats and technical replicates are described in the method description of each experiment above and in figure legends. Standard deviation and *p* value were calculated using the standard deviation “std” and two-sample Kolmogorov-Smirnov (KS) test “kstest2” functions in MATLAB, respectively.

### Reporting summary

Further information on research design is available in the Nature Portfolio Reporting Summary linked to this article.

### Supplementary information

Supplementary Information

Description of Additional Supplementary Files

Supplementary Data 1

Supplementary Data 2

Supplementary Movie 1

Reporting Summary

## Data Availability

The data that support the findings of this study are available from the corresponding author upon reasonable request. Source data underlying graphs are provided in Supplementary Data 1 and Supplementary Data 2. The sequencing data supporting this study has been submitted to the Gene Expression Omnibus (GEO) database under accession number GSE253222.
