# Supplementary material for: Modular tissue-in-a-CUBE platform to model blood-brain barrier (BBB) and brain interaction
Source: Commun Biol. 2024 Feb 28;7:177. doi: 10.1038/s42003-024-05857-8 (PMC10901775; doi:10.1038/s42003-024-05857-8)
Supplement: Supplementary file 1 — Supplementary Information [file 42003_2024_5857_MOESM1_ESM.pdf]

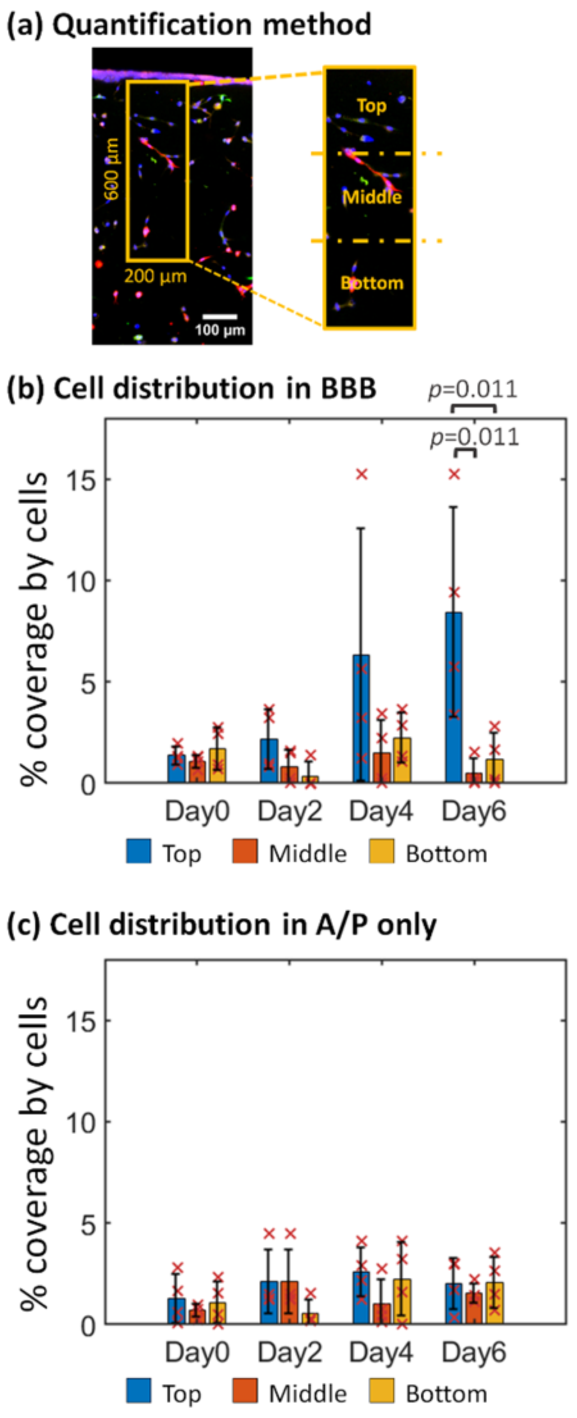

Supplementary Figure 1. Migration of astrocytes and pericytes in Matrigel. (a) For the quantification of astrocyte and pericyte distribution and migration in Matrigel, a region of 600 µm × 200 µm was cropped 20 µm from the border of the Matrigel to exclude the BMEC layer. The cropped region was then divided into 3 sections of 200 µm × 200 µm as the top (0~200 µm), middle (200~400 µm), and bottom (400~600 µm) layers of the sample. (b) In the BBB, cells were uniformly distributed in the Matrigel on days 0 and 2, but by day 4, there were more cells in the top region close to the BMEC, suggesting astrocytes and pericytes elongate and migrate towards the BMEC. (c) In Matrigel with only astrocytes and pericytes without BMEC, the cells remained uniformly distributed in the Matrigel throughout the 6 days of culture, suggesting that astrocytes and pericytes do not self-organise without stimulation by BMEC cues. Bar graph shows average, error bars show standard deviation, and *p* value was calculated by Kolmogorov-Smirnov (KS) test. Blue = Top, Orange = Middle, and Yellow = Bottom.

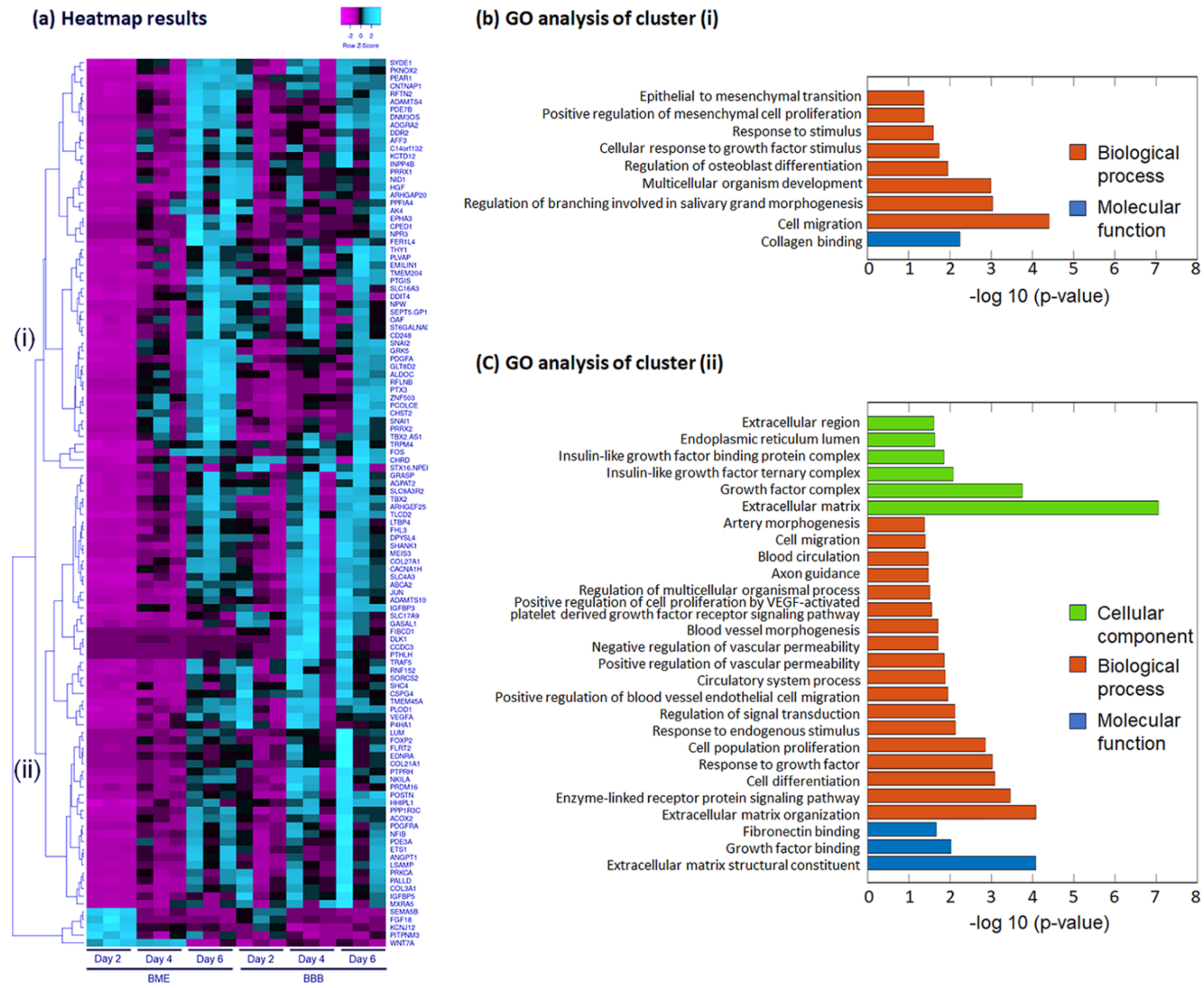

Supplementary Figure 2. RNA-sequencing and GO analysis of BBB and BMEC only. (a) Heatmap of RNA-seq results of BBB and BMEC only on days 2, 4, and 6 showing clustering of genes that (i) increase with increasing culture time, and (ii) are more highly expressed in BBB compared to BMEC only. (b, c) GO analysis of clusters (i) and (ii) suggest that, with increasing culture time, astrocytes and pericytes contribute to cellular migration as well as ECM and vascular remodelling activities. Orange = Biological process, Blue = Molecular function, and Green = Cellular component.

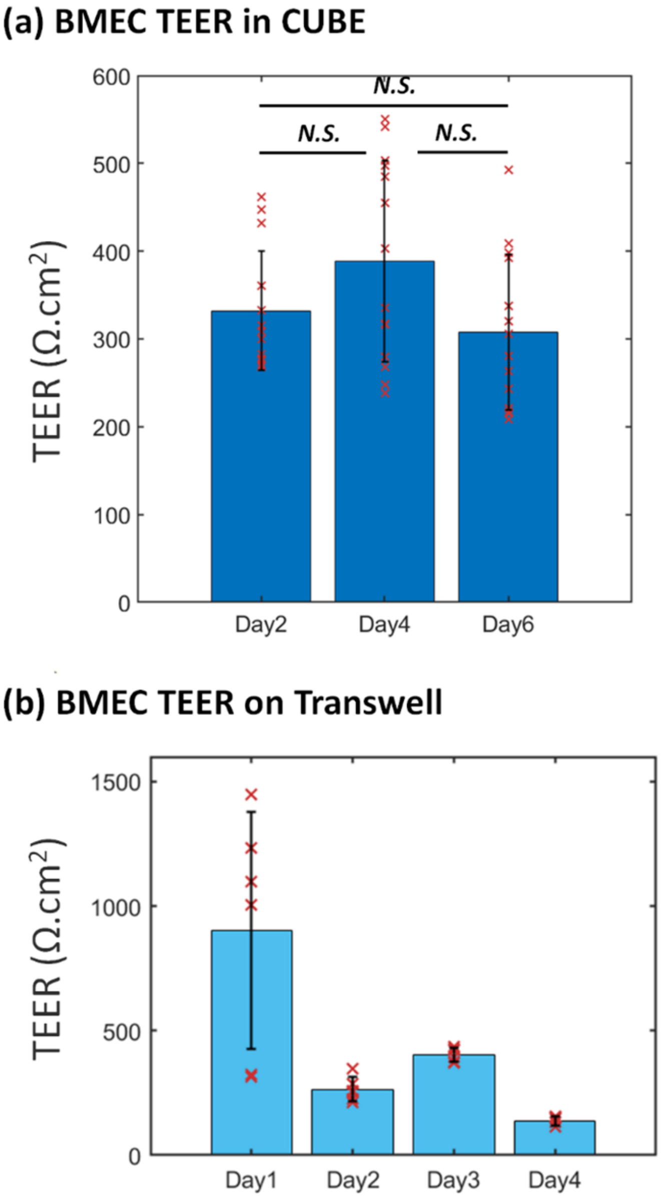

Supplementary Figure 3. TEER of iPSC-derived BMEC in CUBE and on Transwell. (a) BMEC seeded in CUBE with Matrigel only did not show significant differences between culture days 2, 4, and 6. (b) BMEC were seeded on 6.5 mm Transwell with EC+AGM medium, and the resistance measured on the same sample every day for 4 days. Calculated TEER showed a peak of 904 ± 477 $\Omega.cm^2$, which is comparable to reported values after adjustment for differences in insert size. Bar graph shows average and error bars show standard deviation.

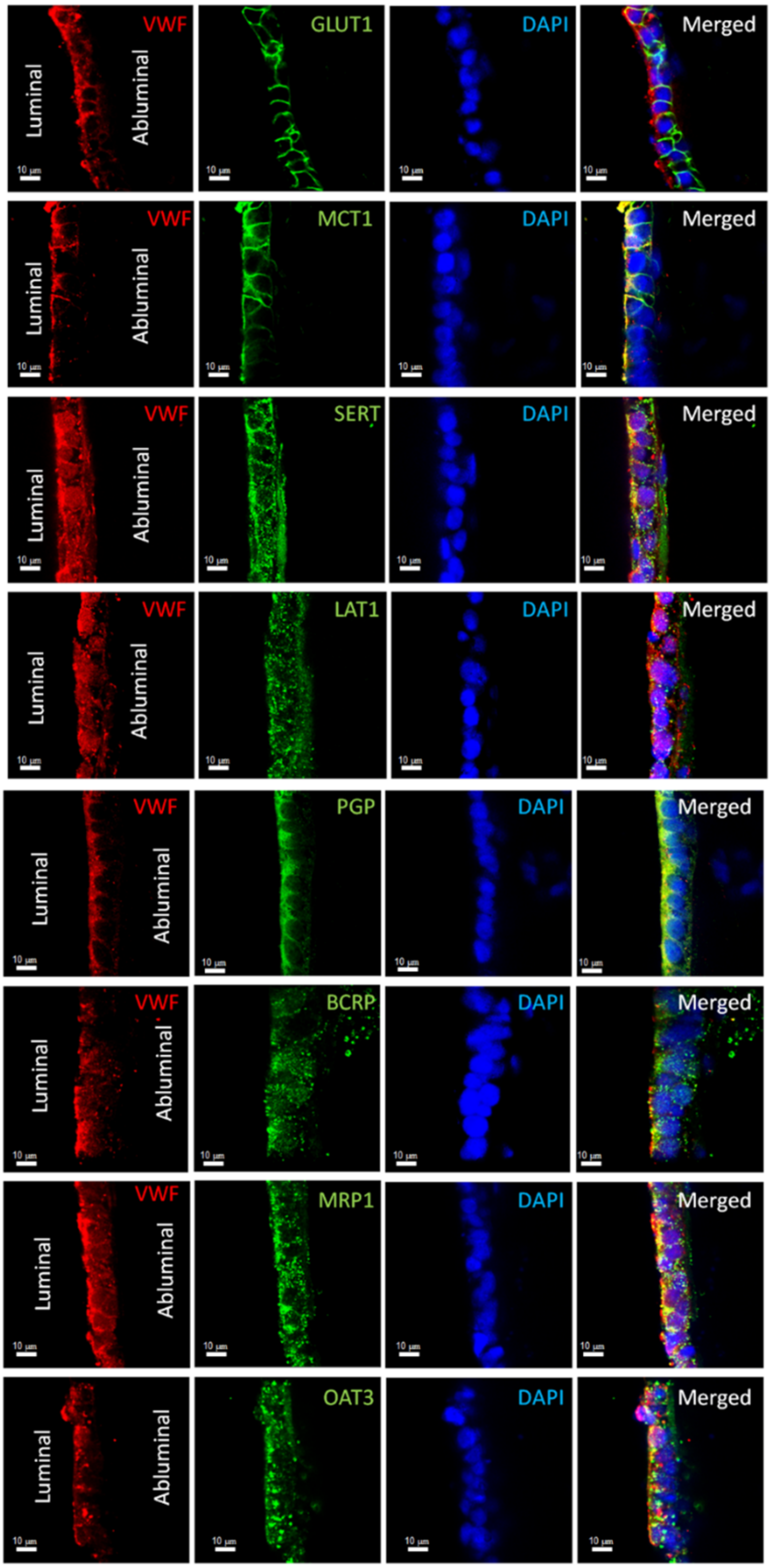

Supplementary Figure 4. Luminal/abluminal view of transporter proteins. Immunofluorescence staining of BMECs from the side view of the BBB show the localisation of transporter proteins in relation to the basement membrane. The efflux transporters PGP, BCRP, MRP1, and OAT3 show higher expressions on the luminal (blood) side, while GLUT1, MCT1, SERT, and LAT1 were expressed on both luminal and abluminal (brain) sides of the BMECs. Optical zoom = 5; Scalebar = 10 µm.

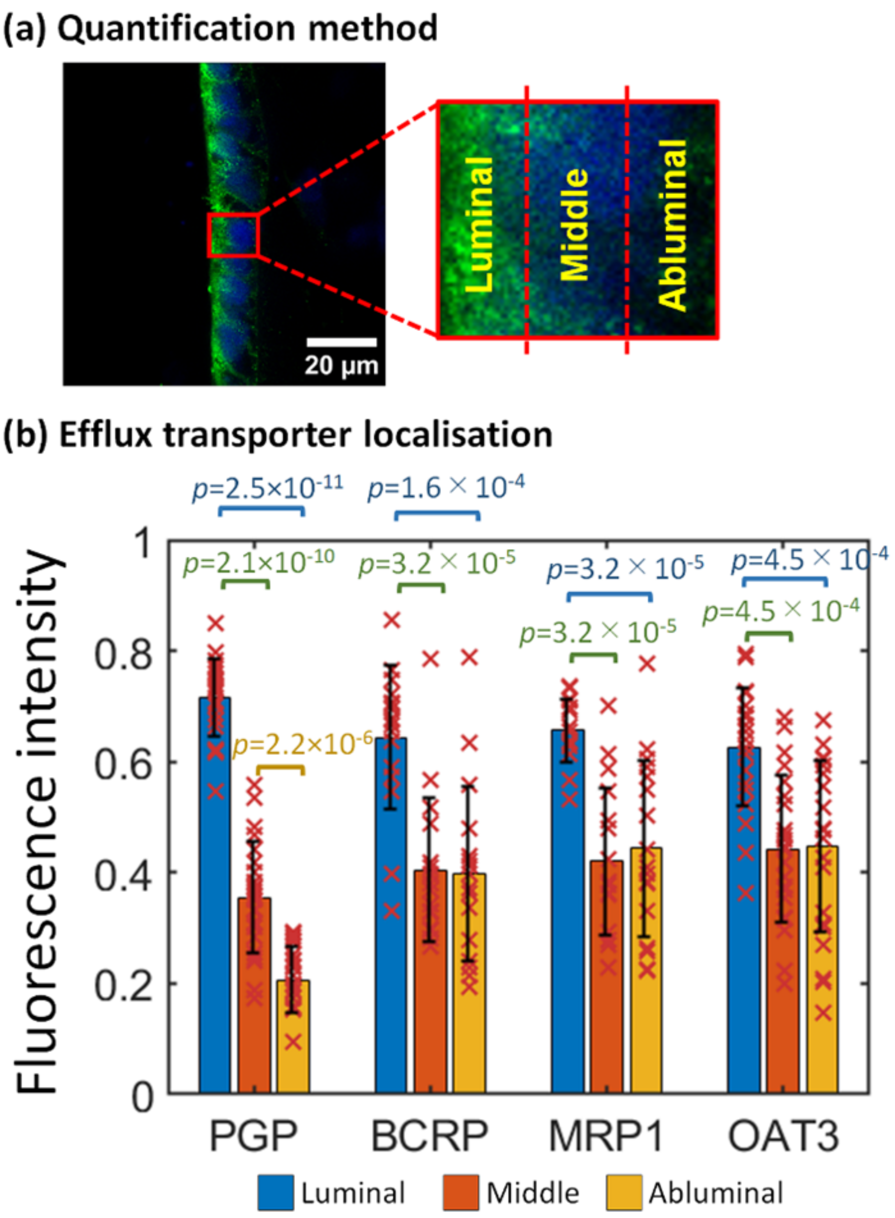

Supplementary Figure 5. Quantification of efflux transporter localisation. (a) To quantify localisation of efflux transporters, an area of a cell was cropped and the transporter intensity across the cropped area was measured. Each cell image was divided into 3 regions of equal size (Luminal, Middle, and Abluminal) and the average intensity of each region calculated. Scalebar = 20 μm. (b) Fluorescence intensity measurement of PGP, BCRP, MRP1, and OAT3 transporter immunofluorescence staining at the luminal, middle, and abluminal regions of BMECs showed higher expression of transporter proteins at the luminal side compared to the abluminal side. Analysis was performed on a total of 5 technical replicates from 2 independent experiments, and 3~5 cell images were analysed from each replicate. Bar graph shows average, error bars show standard deviation, and *p* value was calculated by Kolmogorov-Smirnov (KS) test. Blue = Luminal, Orange = Middle, and Yellow = Abluminal.

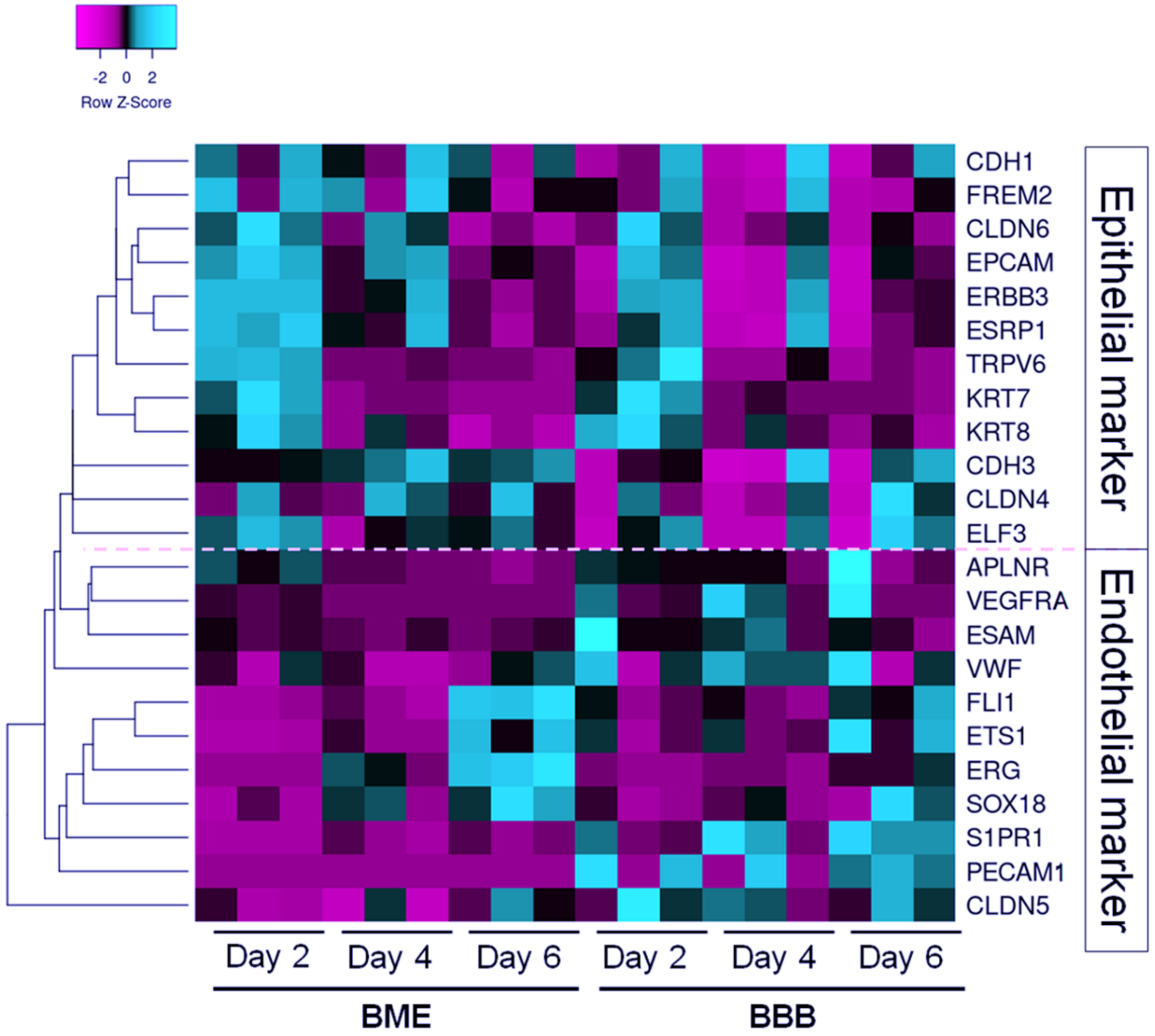

Supplementary Figure 6. Endothelial and epithelial markers extracted from RNA-seq results. Heatmap of endothelial and epithelial markers of BBB and BMEC only show increasing endothelial signature and decreasing epithelial signature with increasing culture time, indicating that culture in the appropriate microenvironment (in a basement membrane, and in co-culture with astrocytes and pericytes) may contribute to BBB endothelial maturation. Magenta = low expression and Cyan = high expression.

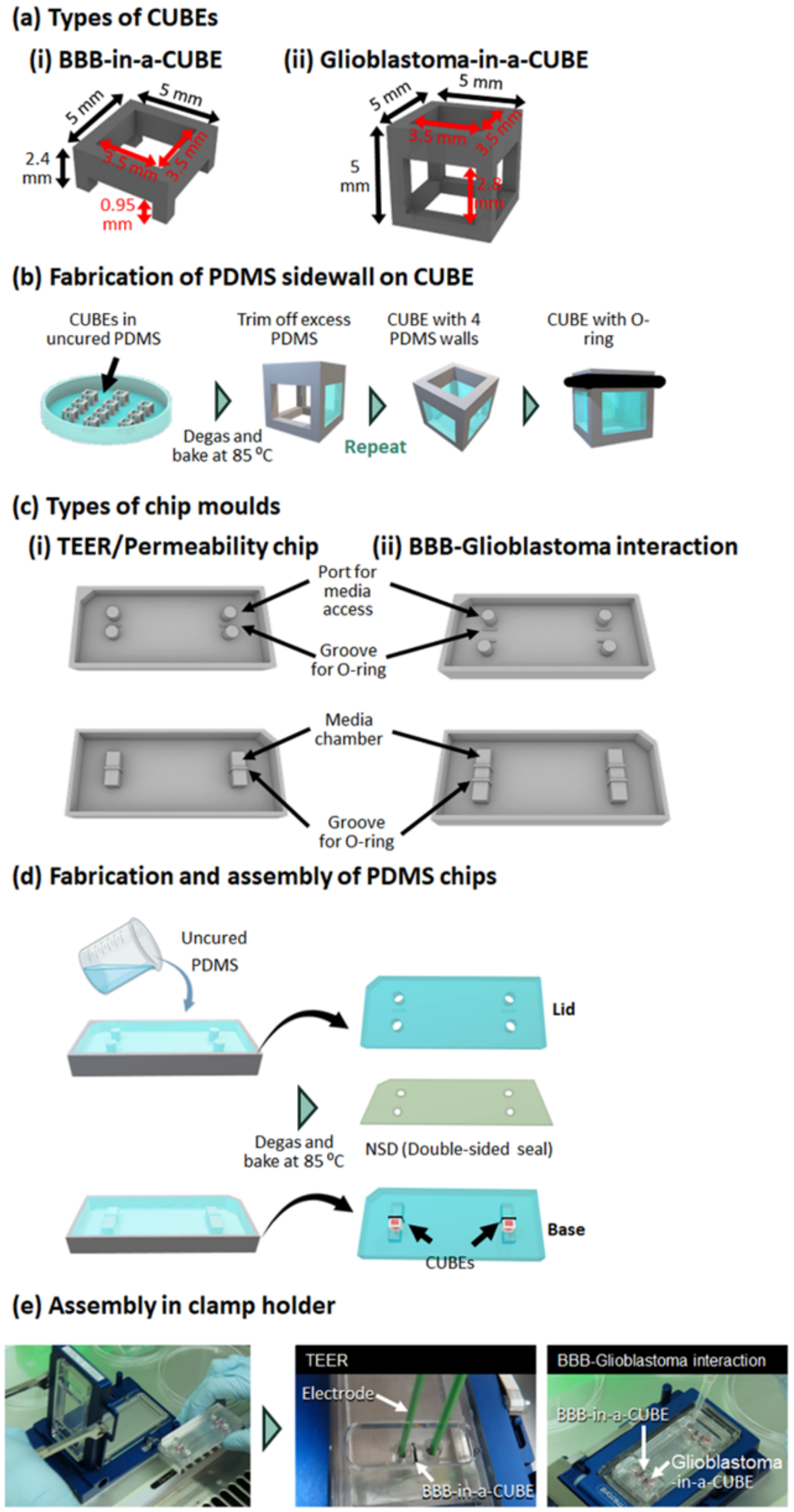

Supplementary Figure 7. Schematic diagram of fabrication and methods processes. (a) Two different types of CUBEs were used in this study: (i) BBB-in-a-CUBE and (ii) Glioblastoma-in-a-CUBE with different dimensions to suit the different types of tissues being reconstructed. (b) Process to adhere PDMS sidewalls to CUBE device. (c) Two different types of chips were used in this study: (i) chip for TEER and permeability measurements and (ii) chip for BBB-Glioblastoma interaction. (d) Process to fabricate and assemble the PDMS chips. (e) Method to assemble the PDMS chips in a clamp holder for TEER or BBB-Glioblastoma interaction.

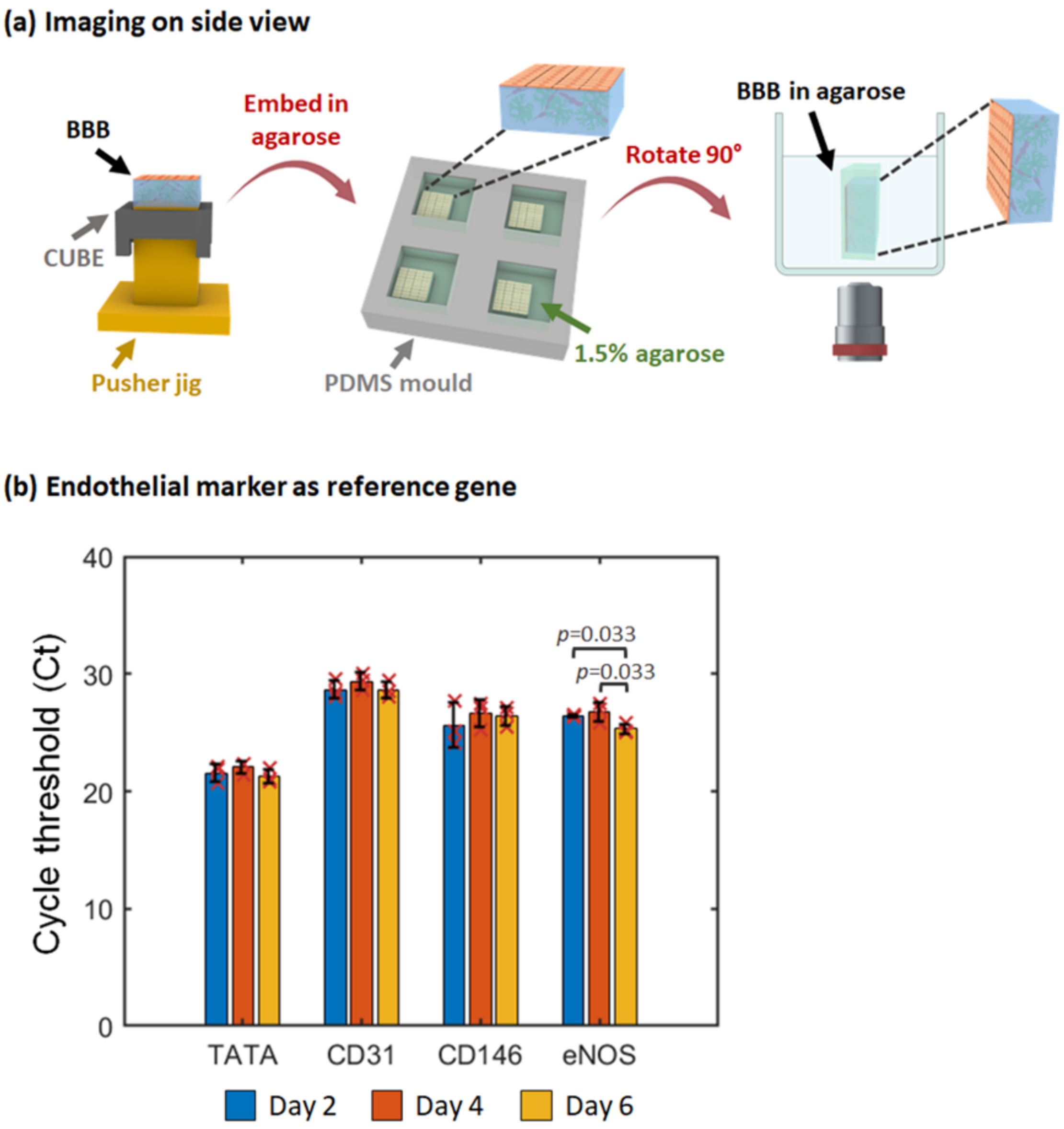

Supplementary Figure 8. Schematics of methods. (a) To image BBB on its side, the BBB is removed from the CUBE by pushing it out using a 3D-printed pusher jig. The sample is then embedded in 1.5% agarose in a PDMS mould. After the agarose has cured, the sample is taken out of the mould and rotated 90° in the imaging dish so that the BBB is on its side. (b) Cycle threshold (Ct) values of *TATA* and endothelial markers *CD31*, *CD146*, and *eNOS* from qPCR was used to identify a suitable reference gene for BMEC. Bar graph shows average, error bars show standard deviation, and p value was calculated by Kolmogorov-Smirnov (KS) test. Blue = Day 2, Orange = Day 4, and Yellow = Day 6.

Supplementary Movie 1: Experimental procedure of Modular-Tissue-in-a-CUBE platform.
