# Supplementary material for: Modular tissue-in-a-CUBE platform to model blood-brain barrier (BBB) and brain interaction
Source: Commun Biol. 2024 Feb 28;7:177. doi: 10.1038/s42003-024-05857-8 (PMC10901775; doi:10.1038/s42003-024-05857-8)
Supplement: Supplementary file 2 — Description of Additional Supplementary Files [file 42003_2024_5857_MOESM2_ESM.pdf]

Description of Additional Supplementary Files

File name: Supplementary Data 1
Description: The source data behind the graphs in the paper.

File name: Supplementary Data 2
Description: RNA-seq data.

File name: Supplementary Movie 1
Description: Experimental procedure of Modular-Tissue-in-a-CUBE platform.
